# Excitable Rho dynamics control cell shape and motility by sequentially activating ERM proteins and actomyosin contractility

**DOI:** 10.1126/sciadv.adn6858

**Published:** 2024-09-06

**Authors:** Seph Marshall-Burghardt, Rodrigo A. Migueles-Ramírez, Qiyao Lin, Nada El Baba, Rayan Saada, Mustakim Umar, Kian Mavalwala, Arnold Hayer

**Affiliations:** ^1^ Department of Biology, Stewart Biology Building, McGill University, Montréal, Québec H3A 1B1, Canada.; ^2^ Graduate Program in Biology, McGill University, Montréal, Québec, Canada.; ^3^ PhD Program in Quantitative Life Sciences, McGill University, Montréal, Québec, Canada.

## Abstract

Migration of endothelial and many other cells requires spatiotemporal regulation of protrusive and contractile cytoskeletal rearrangements that drive local cell shape changes. Unexpectedly, the small GTPase Rho, a crucial regulator of cell movement, has been reported to be active in both local cell protrusions and retractions, raising the question of how Rho activity can coordinate cell migration. Here, we show that Rho activity is absent in local protrusions and active during retractions. During retractions, Rho rapidly activated ezrin-radixin-moesin proteins (ERMs) to increase actin-membrane attachment, and, with a delay, nonmuscle myosin 2 (NM2). Rho activity was excitable, with NM2 acting as a slow negative feedback regulator. Strikingly, inhibition of SLK/LOK kinases, through which Rho activates ERMs, caused elongated cell morphologies, impaired Rho-induced cell contractions, and reverted Rho-induced blebbing. Together, our study demonstrates that Rho activity drives retractions by sequentially enhancing ERM-mediated actin-membrane attachment for force transmission and NM2-dependent contractility.

## Teaser

Rho-enhanced actin-membrane attachment through ERM protein activation drives contractile cell shape changes.

## INTRODUCTION

In response to external stimuli, cells can polarize for directed migration, forming distinct cytoskeletal structures specific to the cell front and back. In the absence of directional cues, many adherent cell types lose their front-back polarization but remain motile and undergo cycles of random protrusion and retraction, often occurring in a wave-like manner (*1*–*4*). This intrinsic cellular behavior occurs on the timescale of minutes and is also widely observed in migrating cells, both in two-dimensional (2D) and in three-dimensional (3D) environments (*4*–*8*). The small guanosine triphosphatases (GTPases) of the Rho family (RhoGTPases), particularly the Rac, Cdc42, and Rho subfamilies (referred to as Rac, Cdc42, and Rho hereafter), regulate actin cytoskeletal dynamics in motile cells by driving protrusive and contractile cell shape changes. Across many cell types and contexts, Rac and Cdc42 have well-defined roles in cellular protrusions, where they promote actin polymerization-driven cell edge extensions (*9*–*13*). Although widely accepted as the master regulator of cellular contractility through its effectors, Rho-associated kinase (ROCK) and nonmuscle myosin 2 (NM2) (*14*, *15*), Rho’s role in regulating cell motility remains incompletely understood. Elevated Rho activity has been observed in cell retractions (*16*–*21*) but also near the cell edge in protrusions and ruffles, where it showed high correlations with local outward cell edge movements (*13*, *22*–*25*). A plausible effector downstream of Rho that drives protrusions is the formin mDia1 (*13*, *26*). However, exogenous activation of Rho causes cells to contract, and subcellular activation of Rho using optogenetics generally induces retractions (*27*–*31*). The ezrin-radixin-moesin proteins (ERMs) are integral components of the cell cortex. When activated, they serve as linkers between the plasma membrane and membrane-proximal actin filaments (*32*). ERMs are activated by the kinases SLK (Ste20-like kinase) and LOK (lymphocyte-oriented kinase), which have recently been identified as Rho effectors (*33*–*36*). Therefore, important emerging questions are whether Rho dynamically controls ERM activation in motile cells undergoing protrusions and retractions, how ERM activation is spatiotemporally coordinated with NM2 activation downstream of Rho, and whether ERM activation regulates cell shape changes.

To address these questions, we visualized Rho activity along with the Rho effectors NM2 or ERMs using fluorescence-based reporters in single unpolarized endothelial cells that exhibit random cycles of protrusions and retractions, and analyzed their spatiotemporal relationships. We found that Rho was enriched in retractions, but not in protrusions, and edge-proximal Rho dynamics were pulsatile, displaying hallmark characteristics of excitability. Rho activation led to the sequential (i) SLK/LOK-mediated activation of ERMs to enhance actin-membrane attachment and (ii) ROCK-dependent NM2 activation to generate local contraction. Activated ERMs coincided with Rho in retractions, whereas NM2 accumulated with a delay. The inhibition of either ROCK or SLK/LOK kinases resulted in elongated cell phenotypes and impaired Rho-induced cell contractility, demonstrating that both ERMs and NM2 are required for Rho-driven contractile cell shape changes.

## RESULTS

### Sparsely plated HT-HUVEC exhibit random, minute-scale protrusion-retraction cycles

**Fig. 1.**
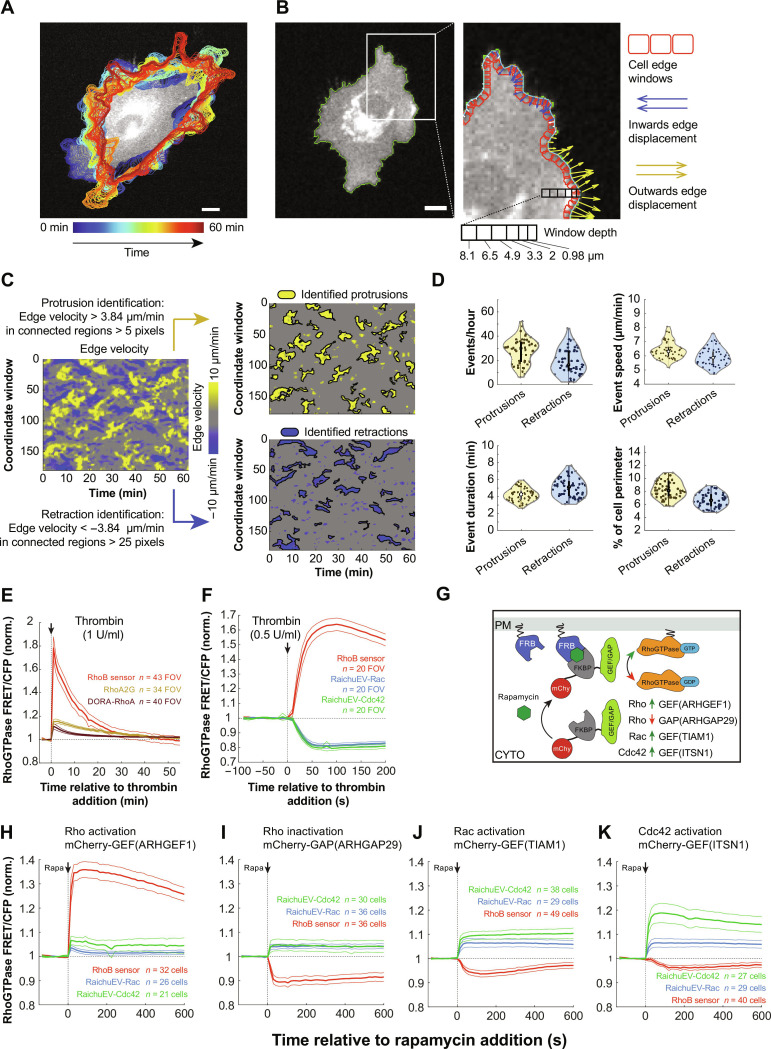
Random motility of HT-HUVEC, cell-edge velocity tracking, and validation of FRET probes for Rho, Rac, and Cdc42 in HT-HUVEC. (**A**) HT-HUVEC cell expressing the RhoB sensor, overlayed with temporally color-coded cell outlines to illustrate protrusion-retraction cycles over 60 min. Scale bar, 10 μm. (movie S1). (**B**) Illustration of cell edge velocity analysis (Materials and Methods). Left: Masked HT-HUVEC cell expressing RhoB sensor. Scale bar, 10 μm. Right: Close-up showing equally spaced cell edge windows (red) and per frame displacement (blue, yellow arrows). (**C**) Left: Corresponding cell-edge velocity map from (A), (top right) segmented protrusions and (bottom right) retractions, based on defined spatiotemporal parameters (Materials and Methods). (**D**) Spatiotemporal parameters of individual protrusions and retractions as identified in (C). Data points are individual events, and violin plot displays means ±25th/75th percentile of events as bolded black lines from *n* = 54 cells from three biological replicates. (**E**) FRET probes for Rho (RhoB sensor, DORA-RhoA, and RhoA2G) were stably expressed in HT-HUVEC, and their responses to thrombin (1 U/ml) were measured over 55 min in subconfluent cultures. Responses normalized to control-treated cells, means of *n* = number of FOV analyzed as indicated ±95% confidence interval (CI), from two biological replicates. (**F**) Responses of the RhoB sensor and FRET probes for Rac and Cdc42 (RaichuEV-Rac and RaichuEV-Cdc42) to thrombin stimulation (0.5 U/ml) were assessed, similar to (E). Means of *n* = 20 fields of view (FOV) per condition ±95% CI, from two biological replicates. (**G**) Strategy to acutely (in)activate RhoGTPases by plasma membrane translocation of RhoGEF and RhoGAP domains through addition of rapamycin. (**H** to **K**) HT-HUVEC expressing RhoB sensor, RaichuEV-Rac, or RaichuEV-Cdc42 were transiently contransfected with Lyn-FRB and mCherry-FKBP-RhoGEF/GAP as indicated, and RhoGTPase FRET/CFP signals were measured in response to rapamycin addition (0.5 μM). Means ±95% CI, normalized to untransfected cells, from *n* = number cells as indicated, from two biological replicates.

In the absence of directional cues, HT-HUVEC (hTERT immortalized human umbilical vein endothelial cells (*29*)) exhibit random motile behavior when plated on uniform substrates at low density. This random motility is characterized by cycles of protrusions and retractions that occur on a timescale of minutes (Fig. 1A and movie S1). We found that this behavior was particularly suitable for quantitative analysis when observed within a few hours after cell plating, when cells had completed spreading and assumed relatively homogenous and compact adhesion surfaces while showing active protrusion-retraction behavior. We quantified cell shape changes based on time-lapse sequences of cells stably expressing fluorescence-based reporters, automated cell segmentation, followed by edge velocity tracking, using established methods (*13*, *17*). For cell edge velocity tracking, segmented cell outlines were divided into 180 equally spaced peripheral coordinate windows spanning the cell perimeter. Each window had an adjustable depth toward the cell interior in which fluorescence-based reporter activity could be measured (Fig. 1B). Window velocities were calculated using per frame displacements, resulting in 2D spatiotemporal edge velocity maps (Fig. 1, B and C). From these maps, we identified protrusions and retractions as events exceeding defined minima of outward and inward velocity and spatiotemporal scale (Fig. 1C and Materials and Methods). This analysis revealed that over a 1 h duration, randomly sampled cells (*n* = 54) had, on average, 27.63 ± 10.27 (means ± SD) individual protrusion events, each with an average mean velocity of 6.39 ± 0.61 μm/min, lasting for 4.21 ± 0.75 min and spanning 8.26 ± 1.32% of the cell perimeter. In comparison, the same cells had 20.79 ± 12.17 retraction events with a mean velocity of −5.93 ± 0.63 μm/min, lasting for 5.03 ± 1.10 min and spanning 6.69 ± 1.10% of the cell perimeter (Fig. 1D).

### Spatiotemporal analysis of Rho, Rac, and Cdc42 using FRET-based activity probes in randomly motile HT-HUVEC

To enable spatiotemporal analysis of Rho, Rac, and Cdc42 activities in thresholded protrusions and retractions, we first tested the suitability of existing fluorescence resonance energy transfer (FRET)–based activity probes in HT-HUVEC. For Rho, we chose RhoA-based RhoA2G (*37*), DORA-RhoA (*38*, *39*), and a RhoB-based RhoB sensor (*18*). The probes were stably expressed in HT-HUVEC via lentiviral transduction, and their responses to acute, pathway-selective perturbations were assessed (Materials and Methods). Activation of Rho using thrombin resulted in increased Rho activity as reported by all three probes, with the RhoB sensor showing by far the strongest response (Fig. 1, E and F). RhoA and RhoB share >85% sequence identity, and functionally many guanine nucleotide exchange factors (GEFs), GTPase activating proteins (GAPs), and downstream effectors (*33*, *40*). RhoA and RhoB differ in their C-terminal domains, resulting in distinct lipid modifications and localization patterns, with RhoA and RhoB being mainly cytoplasmic and membrane localized, respectively (*41*, *42*). Consistent with this, the RhoA-based probes RhoA2G and DORA-RhoA primarily localized to the cytoplasm, whereas the RhoB sensor was mainly plasma membrane–localized (fig. S1A). We found that local Rho activities reported by RhoA2G and DORA-RhoA appeared to be affected by the local cell geometry when viewed by epifluorescence microscopy. The highest activities were generally enriched in the cell periphery and lowest activities in the cell center, corresponding to high and low membrane/cytoplasm ratios, respectively [fig. S1A (a and b)]. To assess a possible localization bias of reported Rho activities, we performed pixel-by-pixel correlations between normalized Rho FRET/cyan fluorescent protein (CFP) activity ratios and their localizations for each of the three probes. RhoA2G and DORA-RhoA both yielded significantly stronger anticorrelations compared to the RhoB sensor [fig. S1A (c) and B], indicating that the activities reported by RhoA-based probes were affected by local cell geometry. Thus, ratiometric FRET analysis based on epifluorescence imaging data insufficiently corrects for cell geometry when most of the inactive probe resides in the cytoplasm, as in the case of RhoA-based probes. To further explore the suitability of the RhoB sensor, we tested its response to plasma membrane translocation of mCherry-FKBP-ARHGEF1(GEF) and mCherry-FKBP-ARHGAP29(GAP) through rapamycin-induced heterodimerization with plasma membrane–localized Lyn_11_-FRB to acutely (in)activate Rho activity in cells (Fig. 1, G to I). ARHGEF1 and ARHGAP29 are a Rho-specific GEF and GAP, respectively, and the RhoB sensor showed bidirectional, expected responses to plasma membrane translocation of their catalytic domains. Because of the superior response to thrombin, the absence of a noticeable localization bias, and the robust responses to both activating and inactivating Rho-regulators, we considered the RhoB sensor as the best suited Rho probe for spatiotemporal analysis in HT-HUVEC.

As Rac and Cdc42 reporters, we chose RaichuEV-Rac and RaichuEV-Cdc42 (*17*, *43*–*45*). They reported expected responses to pathway-stimulating signals. Increased activities occurred upon plasma membrane translocation of mCherry-FKBP-TIAM1(GEF) (Fig. 1J) and mCherry-FKBP-ITSN1(GEF) (Fig. 1K) with TIAM1 being a Rac specific and ITSN1 a Cdc42-specific RhoGEF. We noted that RaichuEV-Rac and RaichuEV-Cdc42 co-activated in response to the GEF translocations, consistent with positive feedback mechanisms that engage during cell protrusions (*46*). RaichuEV-Rac and RaichuEV-Cdc42 were inactivated and activated upon Rho activation and inactivation, respectively (Fig. 1, F and I) (with the exception for ARHGEF1-activated Rho, Fig. 1H), consistent with an antagonism between protrusive and contractile RhoGTPase activities (*47*). In addition to expected bidirectional responses of all RhoGTPase probes tested, their temporal responses were on the order of seconds, further validating their suitability for spatiotemporal analysis in motile cells.

**Fig. 2.**
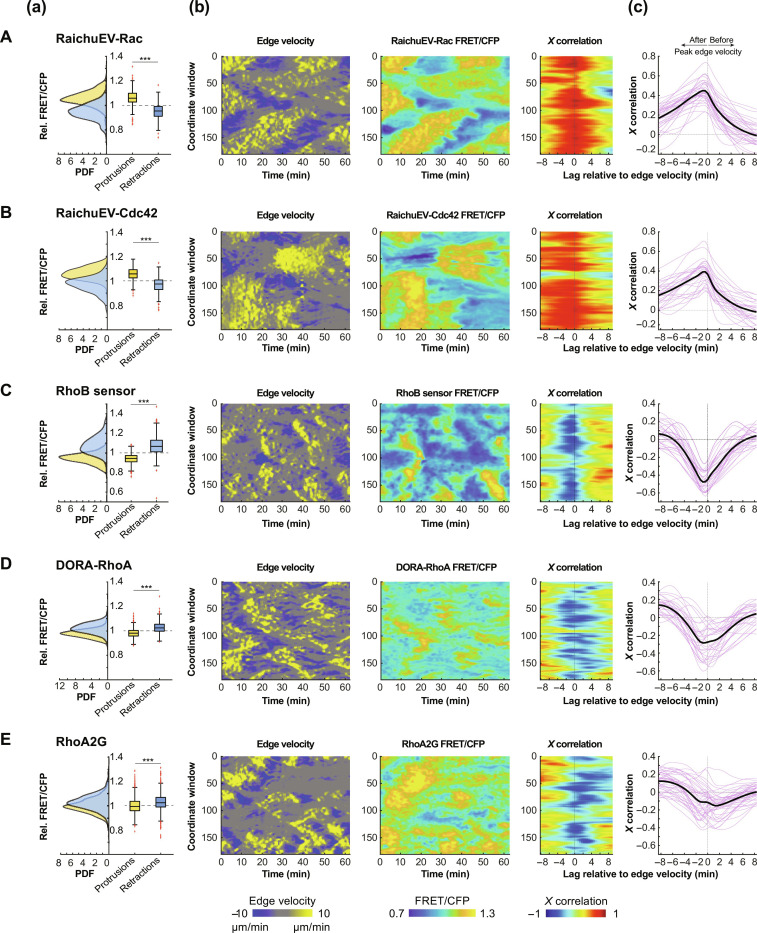
Spatiotemporal analysis of Rac, Cdc42, and Rho activities in HT-HUVEC. (**A**) RaichuEV-Rac. (**B**) RaichuEV-Cdc42. (**C**) RhoB sensor. (**D**) DORA-RhoA. (**E**) RhoA2G (movies S2 to S6). (a) Graphs showing differences in relative FRET activity in thresholded protrusions and retractions for each probe tested at a window edge depth of 1.95 μm. Protrusions > 3.86 μm/min, retractions < −3.86 μm/min, each minimum 25 pixels on edge velocity heatmaps. Individual protrusions and retractions were normalized to edge-proximal FRET activity in nonmoving edge areas during the same time span. Left *x* axis shows probability density functions (PDF) of retractions in blue and protrusions in yellow. Box plot on the right *x* axis: the bolded center line denotes dataset median, colored boxes show 25th and 75th percentiles, and whiskers show total dataset range. Outliers are shown with red crosses. Data for each probe were from three biological replicates. RaichuEV-Rac: 30 cells, 489 protrusions, 299 retractions. RaichuEV-Cdc42: 29 cells, 552 protrusions, 308 retractions, RhoB sensor: 36 cells, 726 protrusions, 413 retractions. DORA-RhoA: 30 cells, 771 protrusions, 672 retractions, RhoA2G: 40 cells, 1100 protrusions, 1021 retractions. ***P < 0.001, Mann-Whitney *U* test. (b) Examples of spatiotemporal heatmaps for individual cells from 62.5-min time-lapse acquisitions. Left: Edge velocity, with protrusions in yellow and retractions in blue. Middle: Corresponding RhoGTPase activity, measured within 1.95 μm from the cell edge. Right: Cross-correlation between edge velocity and RhoGTPase activity from the two heatmaps. (c) Cross-correlation of RhoGTPase activity relative to edge velocity for each probe at a window depth of 1.95 μm. Pink traces represent single cells, and bold black traces are means. Negative lag denotes peak RhoGTPase activity following peak edge velocity and vice versa. RaichuEV-Rac, *n* = 34 cells; RaichuEV-Cdc42, *n* = 28 cells; and RhoB sensor, *n* = 27 cells, from two biological replicates. DORA-RhoA, *n* = 29 cells and RhoA2G, *n* = 40 cells, from three biological replicates.

We then imaged randomly motile HT-HUVEC stably expressing RaichuEV-Rac, RaichuEV-Cdc42, RhoB sensor, DORA-RhoA, or RhoA2G at 25-second intervals to identify spatiotemporal activation patterns of Rho GTPases during protrusion-retraction cycles (Fig. 2 and movies S2 to S6). For each probe, the (a) panels show RhoGTPase activity differences between thresholded protrusions and retractions, normalized to nonmoving membrane segments in the same time period. The (b) panels show single-cell spatiotemporal maps visualizing edge velocities and RhoGTPase activities within 1.95 μm from the cell edge and cross-correlation between the two maps. The (c) panels show cross correlations between edge velocity and RhoGTPase activities compiled from multiple cells. As expected, relative to membrane segments classified as nonmoving, Rac1 and Cdc42 were elevated in protrusions and reduced in local retractions [Fig. 2, A and B (a), (b)]. Cross-correlation analysis between edge velocity and Rac1 [Fig. 2A (c)] or Cdc42 [Fig. 2B (c)] showed strong positive correlations with lags of negative 25 to 50 s (i.e., peak RhoGTPase activities lagging peak edge velocities), confirming previous results (*13*, *48*). The RhoB sensor showed an opposite pattern of activity—uniformly inactive in protrusions and variably but consistently activated in retractions [Fig. 2C (a), (b)]. The cross-correlation between edge velocity and RhoB sensor activity yielded a sharply negative peak lagging edge velocity by 25 to 50 s [Fig. 2C (c)]. Similar analyses using DORA-RhoA or RhoA2G confirmed enrichment of Rho activity in retractions [Fig. 2, D and E (a), (b)]. Cross-correlations between edge velocity and RhoA2G or DORA-RhoA signals were less uniform between cells but similarly consistently negative [Fig. 2, D and E (c)], confirming that edge-proximal Rho activity is correlated with local edge retractions and anticorrelated with protrusions. We also compared the activity patterns of the RhoB sensor with a localization-based Rho probe [dTomato-2xrGBD, (*19*)], coexpressed in the same cells, and found that they closely matched (fig. S2 and movie S7). Together, our analyses demonstrate that micrometer and minute-scale cell shape changes in randomly motile HT-HUVEC are associated with distinct RhoGTPase activity patterns, with elevated Rac and Cdc42 activities protrusions and elevated Rho activity in retractions.

### Pulsatile activation of Rho during membrane retractions

**Fig. 3.**
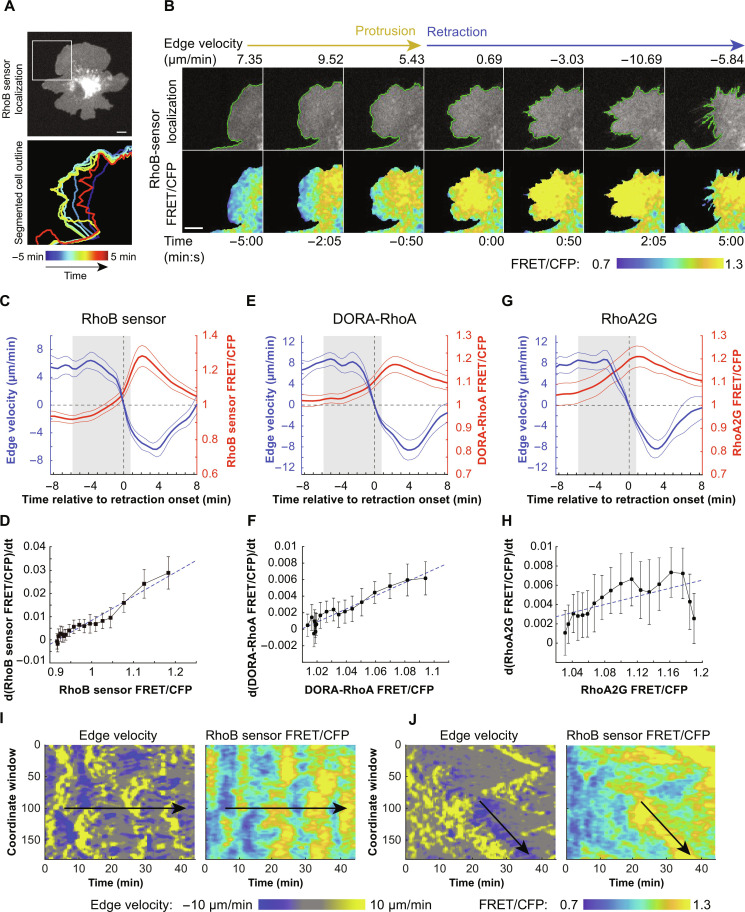
Pulsatile activation of Rho in cell-edge retractions. (**A**) Top: HT-HUVEC expressing the RhoB sensor, with region of interest (ROI) denoted by white box. Scale bar, 10 μm. Bottom: Local cell-edge displacements over 10 min of the protrusion-retraction event shown in (B). (**B**) Time-lapse illustrating RhoB sensor activation during a protrusion-retraction event during a 10-min interval. Time = 00:00 denotes retraction onset. Scale bar, 10 μm. (movie S8). (**C**, **E**, and **G**) Activity buildup plots displaying averaged RhoB sensor, DORA-RhoA, and RhoA2G activities, respectively, compared during protrusion-retraction transitions. Time = 0 denotes the protrusion-retraction transition. Gray shaded region shows time points included in the rate-of-change analysis in (D), (F), and (H). Means ±95% CI. RhoB sensor: *n* = 23 events, DORA-RhoA: *n* = 33 events, RhoA2G: *n* = 25 events, from three biological replicates. (**D**, **F**, and **H**) Plot of the rate of change in Rho activation as a function of Rho activity from approximately *t* = −6 min to *t* = 1 min (18 25-s time points) from the buildup plots for RhoB sensor, DORA-RhoA, and RhoA2G. Black boxes denote time point means, and error bars show ±95% CI. Linear line of best fit shown by dotted blue line. (**I** and **J**) Edge velocity (left) and RhoB sensor activity maps (right) (window depth 1.95 μm) of representative cells illustrating (I) pulsatile activity and (J) a propagating wave of RhoB sensor activity. ROI was denoted by black arrows on both sets of maps (movies S9 and S10).

We subsequently focused on Rho activity in membrane retractions because Rac and Cdc42 have well-documented roles in driving membrane protrusions. Visually, RhoB sensor activity propagated from the cell interior toward the cell edge before retraction onset (Fig. 3, A and B, and movie S8). Our cross-correlation analyses between edge velocities and Rho activity yielded a negative correlation and time lag; however, these parameters result from averaged relationships in protrusions and retractions combined. To specifically determine the kinetics of Rho activation in retractions (but not Rho inactivation in protrusions), we analyzed Rho activity in cell edge regions where protrusions transitioned into retractions (fig. S3 and Materials and Methods). To compare Rho sensor activation across multiple retraction events, we aligned Rho kinetics and edge velocity time courses by setting time = 0 to the transition point from protrusion to retraction (edge velocity = 0). The resulting averaged Rho activity time courses showed a notable, pulse-like activation during retractions for RhoB sensor, DORA-RhoA, and RhoA2G (Fig. 3, C, E, and G). Rho activity began to rise well before retraction onset, while edge velocity remained positive. Upon reaching the transition point, Rho activity levels were already well above basal activation levels and increased in a nonlinear fashion. Unexpectedly, Rho activity did not remain statically elevated throughout the retraction. Peak Rho activity 2 min after retraction onset was followed by a marked reduction and return to baseline levels, indicating the presence of an inactivation mechanism triggered before retraction completion.

Such pulse-like patterns of activity are generated by activator-inhibitor coupled excitable systems, composed of fast autoactivation paired with delayed self-inhibition (*49*–*51*). Plotting the rate of change of Rho activity as a function of Rho activity during the buildup phase (approximately 6 min before retraction onset to 1 min after retraction onset, shaded rectangle in Fig. 3, C, E, and G) revealed a linear positive correlation (Fig. 3, D, F, and H), i.e., Rho activity increase accelerating with increasing Rho activity. This is consistent with active Rho engaging in a positive feedback loop to further increase its own activation, a phenomenon noted in other instances of excitable Rho behavior (*52*, *53*). The smaller peak amplitudes and weaker positive correlations that we observed when using the DORA-RhoA and RhoA2G probes compared with the RhoB sensor were likely due to aforementioned reduced dynamic range and cell geometric bias artificially increasing Rho activity near the cell edge (fig. S1). In addition, we observed pulsatile behavior (Fig. 3I, Movie S9) and propagating waves (Fig. 3J, Movie S10) of both Rho activity and edge retraction, patterns consistent with an excitable Rho signaling network. Buildup profiles of Rac and Cdc42 activity in protrusion-retraction transitions showed a decrease in activity aligned with retraction onset but no pulsatile patterning (fig. S4). Thus, using three different FRET probes, we found that Rho activity increases before retraction onset and is pulsatile, with rapid activation during and inactivation before the retraction process is completed.

### Rho, ROCK, and NM2 are part of an excitable system during membrane retractions

**Fig. 4.**
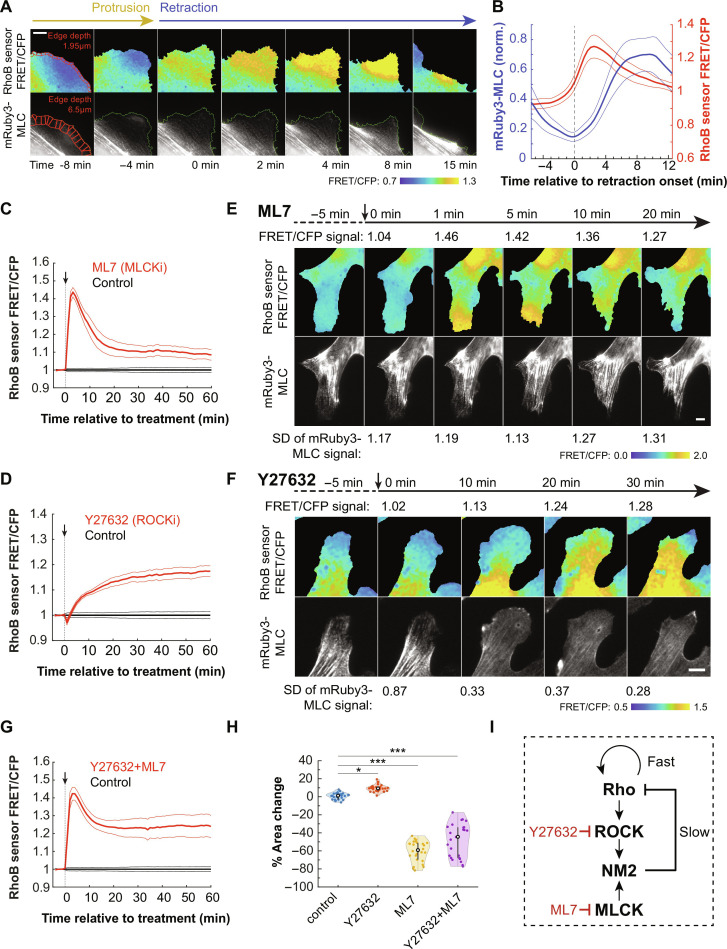
Rho, ROCK, and NM2 are part of an excitable system during membrane retractions. (**A**) Time-lapse showing Rho activation and NM2 accumulation in a RhoB sensor and mRuby3-MLC–expressing HT-HUVEC. Red edge windows show 1.95-μm window depth for RhoB sensor and 6.5 μm for MLC-mRuby3. *t* = 0 denotes retraction onset. Scale bar, 10 μm. (movie S11). (**B**) Thirty protrusion-retraction events from three biological replicates. RhoB sensor window depth, 1.95 μm; mRuby3-MLC window depth, 6.5 μm. Bolded lines represent means, bordered by ±95% CI. (**C**, **D**, and **G**) RhoB sensor activity responses to acute inhibition of MLCK (ML7, 20 μM), ROCK (Y27632, 20 μM), or ROCK and MLCK (Y27632 + ML7, both 20 μM). Drug responses were normalized to control-treated samples. Bolded lines denote means, bordered by ±95% CI. Twenty-six FOV for each condition, from three biological replicates. (**E**) Effect of MLCK inhibition (ML7, 20 μM) on RhoB sensor activity (top) and mRuby3-MLC distribution (bottom). RhoB sensor FRET/CFP signal labeled above top row, and standard deviation of pixel distribution to measure granularity of MLC signal labeled below bottom row. Scale bar, 10 μm. (movie S12). (**F**) Effect of ROCK inhibition (Y27632, 20 μM) on RhoB sensor activity (top) and mRuby3-MLC distribution (bottom). RhoB sensor activity labeled above top row, and SD of pixel distribution to measure granularity of mRuby3-MLC signal labeled below bottom row. Scale bar, 10 μm. (movie S13). (**H**) Percent area change of cell adhesion surface 30 min after addition of control, 20 μM Y27632, 20 μM ML7, or a combination of 20 μM Y27632 and 20 μM ML7. Bolded circles denote median, black lines denote 25th/75th percentiles, and individual data points for *n* = 26 FOV each for control, Y27632, and ML7 conditions, from three biological replicates. *P < 0.05 and ***P < 0.001, one-way ANOVA/Tukey-Kramer. (**I**) Diagram illustrating the relationship between Rho’s downstream effectors ROCK and NM2 and the drugs used in (C) to (H).

Biological excitability is characterized by positive self-feedback that generates a rapid increase in activity, followed by delayed inhibition by a downstream effector that returns activity to basal levels (*50*, *51*). Excitable signaling modules can drive cell protrusions during chemotaxis or propagating waves of actin polymerization (*17*, *50*, *54*–*56*). Moreover, previous studies have found that Rho and its cytoskeletal effectors are excitable, resulting in pulsatile or wave-like propagation of Rho activity (*52*, *53*, *57*, *58*). Several studies identified that accumulation of the downstream target NM2 with a delay relative to Rho can act as a negative regulator of Rho through recruitment of RhoGAPs (*52*, *53*, *57*–*59*). To test whether this was the case during membrane retractions, we first coexpressed fluorescently tagged myosin regulatory light chain (mRuby3-MLC) and RhoB sensor in HT-HUVEC to analyze NM2 activation alongside edge velocity and Rho activity. The localization-based NM2 activity reporter mRuby3-MLC is cytoplasmic when inactive but forms distinct puncta when incorporated into myosin filaments upon activation. Edge-proximal mRuby3-MLC signal was low during protrusions and early retractions (Fig. 4A and movie S11). Levels increased in intensity beginning at retraction onset, peaking several minutes after maximal Rho activity levels (Fig. 4B). Cross-correlation analysis between RhoB sensor and mRuby3-MLC signal showed a delayed positive correlation (fig. S5A), significantly highest at edge depths of 5 to 8 μm (fig. S5, B and C), reflecting the absence of edge-proximal myosin and localization to inward moving actin filaments. The slow, delayed accumulation of myosin following Rho activation in protrusion-retraction events closely matched the localization of negative regulators in excitable system models (*50*), consistent with NM2 activation downstream of Rho activity being part a negative regulator of Rho itself.

To test this further, we measured RhoB sensor activity in response to acute treatments of cells with inhibitors of ROCK (Y27632, 20 μM) and myosin light-chain kinase (MLCK) (ML7, 20 μM), i.e., kinases acting upstream of NM2 activation. We were unable to use blebbistatin as a direct inhibitor of myosin motor activity, as the color of solutions containing blebbistatin interfered with FRET-based Rho activity measurements. Consistent with NM2 acting as a negative regulator of Rho, both drug additions resulted in increased RhoB sensor activity, although with distinct kinetics. ML7 addition resulted in a sharp, transient increase (Fig. 4C), whereas Y27632 addition caused a slower but persistent increase of Rho activity (Fig. 4D). Within MLCK-inhibited cells, stress fibers were maintained or increased, and RhoB sensor activity transiently increased in peripheral regions (Fig. 4E and movie S12), whereas ROCK inhibition caused a sustained increase of RhoB sensor activity throughout the cells, particularly where stress fibers had dissolved, and except for peripheral ruffling regions (Fig. 4F and movie S13). Treating cells with both drugs resulted in a combined response reflective of the two independent pathways of MLC phosphorylation (Fig. 4G). Similar activation patterns were observed using the DORA-RhoA and RhoA2G probes (fig. S6). Consistent with ROCK and MLCK preferentially activating NM2 in the cell interior and the cell periphery, respectively, ROCK inhibition increased and MLCK inhibition decreased the cells’ adhesion surface (Fig. 4H) (*60*, *61*). Together, both inhibitors increased Rho activity, demonstrating generally that activated NM2 negatively regulates Rho. Specifically, the sustained increase in Rho activity following ROCK inhibition and stress fiber dissolution suggested that the Rho-ROCK-NM2 pathway plays a critical role as a negative regulator of excitable Rho activity (Fig. 4I).

### Rho activity and ERM activation are spatiotemporally coupled

**Fig. 5.**
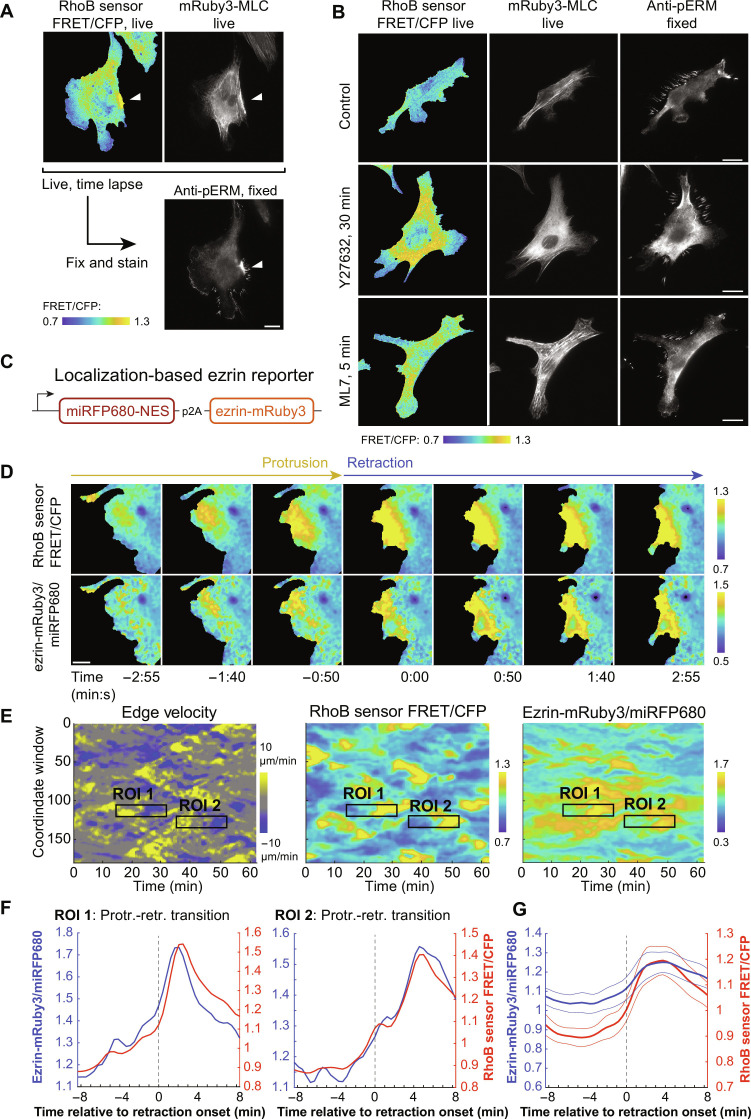
Rho activity and ERM activation are spatiotemporally coupled. (**A**) HT-HUVEC coexpressing RhoB sensor and mRuby3-MLC showing RhoB FRET activity (live), mRuby3-MLC (live), and corresponding anti-pERM signals following fixation and immunostaining. White arrowhead denotes retraction site. Scale bar, 20 μm. (movie S14). (**B**) HT-HUVEC coexpressing RhoB sensor and mRuby3-MLC were imaged after no treatment, 30 min after 10 μM Y27632 addition, or 5 min after 5 μM ML7 addition; fixed; and processed for anti-pERM immunostaining. Drug-induced increases of RhoB sensor activity colocalized with increased pERM staining. Scale bars, 20 μm. (**C**) Schematic of the localization-based ezrin reporter. (**D**) Time-lapse showing RhoB sensor activity and ezrin-mRuby3/miRFP680 ratio during an individual protrusion-retraction transition. *Time* = 0:00 denotes retraction onset. Scale bar, 10 μm. (movie S15). (**E**) Spatiotemporal heat maps depicting edge velocity, RhoB sensor activity, and ezrin-mRuby3/miRFP680 ratio, both measured at 1.95-μm window depths. ROIs 1 and 2 correspond to the regions used to generate the correspondingly labeled individual activity buildup plots in (F). (**F**) Protrusion-retraction events outlined in (E) displaying average ezrin-mRuby3/miRFP680 ratios in blue and averaged RhoB sensor activities in red. *Time* = 0 denotes retraction onset. (**G**) Compiled protrusion-retraction transitions showing normalized ezrin-mRuby3/miRFP680 ratios in blue and RhoB sensor activities in red. *Time* = 0 denotes protrusion-retraction transition. Bolded lines represent means, bordered by ±95% CI. Nineteen events from three biological replicates.

Given that mRuby3-MLC signal was at its lowest near the cell edge at retraction onset and accumulated with a substantial delay relative to Rho (Fig. 4B), we questioned whether additional Rho effectors besides NM2 were involved in driving early phases of membrane retractions. Rho is also known to activate ERMs through the kinases SLK and LOK (*33*, *34*, *36*). Because the release of cortical actin from the plasma membrane is necessary for the initiation of membrane protrusions (*45*, *62*), we reasoned that re-establishment of membrane-cortex attachment through activation of ERMs could be involved in retraction initiation. To test this, we first performed live-cell imaging of RhoB sensor and mRuby3-MLC–expressing HT-HUVEC, then fixed the cells, and stained them using an anti-phospho-ERM (pERM) antibody that detects activated (phosphorylated) ERMs. Matching membrane retractions from time-lapse sequences to pERM signals revealed a notable enrichment of pERM in retractions, consistent with Rho locally activating ERMs (Fig. 5A and movie S14). We also found that in ROCK- and MLCK-inhibited cells, locally increased Rho activity colocalized with locally increased pERM signals, further supporting Rho activity acting upstream of ERM activation (Fig. 5B).

We next sought to monitor the spatiotemporal relationship between Rho and ERM activation in retractions in live cells. We generated a localization-based ERM activation reporter, composed of mRuby3-tagged ezrin and cytoplasmic miRFP680 that we expressed stoichiometrically from the same mRNA using a p2A ribosomal skipping sequence (Fig. 5C). Because ERMs are stabilized in the plasma membrane upon activation and crosslinking with F-actin (*63*, *64*), we reasoned that the per-pixel ratio of ezrin-mRuby3/miRFP680 fluorescence should be an indication of local ezrin and/or ERM activation. To test this, we generated cells stably expressing versions of this reporter containing either wild type (WT) or mutant nonphosphorylatable ezrin (T567A) that cannot act as an actin-membrane crosslinker (*65*). We then treated the cells with low doses of nocodazole (5 μM) to enhance Rho signaling dynamics (*57*), fixed the cells, and stained them using an anti-pERM antibody to assess co-occurrence of locally increased pERM and ezrin-mRuby3/miRFP680 ratios in cells. The WT version of the reporter produced significantly elevated ezrin-mRuby3/miRFP680 ratios that colocalized with patches of elevated pERM compared to the mutant version (fig. S7, A to D). That ezrin-T567A-mRuby3/miRFP680 ratios were slightly elevated in pERM patches as well relative to the whole cell average is likely explained by unphosphorylated ERMs partially localizing to the plasma membrane (*63*, *64*) and the increased surface/volume ratios in the cell periphery, where most pERM patches were observed (fig. S7, A to D). We therefore used ezrin-WT-mRuby3/miRFP680 ratios to monitor ERM activation alongside Rho activity in live cells.

The analysis of cell-edge dynamics, RhoB sensor activity, and ezrin-mRuby3/miRFP680 together revealed a notable spatiotemporal correlation of Rho activity and ezrin-mRuby3/miRFP680 (Fig. 5, D and E, and movie S15). Activity buildup plots of both individual and compiled (Fig. 5, F and G) protrusion-retraction events demonstrated no detectable time lag between Rho activation and ezrin signal in retractions. This tight spatiotemporal correlation between Rho activity and our ezrin reporter suggested that ERMs are immediate-early effectors of Rho, which serve to enhance the force transmission between actin and the plasma membrane during retractions. Because causation cannot be inferred from correlation alone, we investigated, in the following, a possible functional relationship between the activation of Rho and ERMs in retractions.

### Rho rapidly activates ERMs via SLK/LOK

**Fig. 6.**
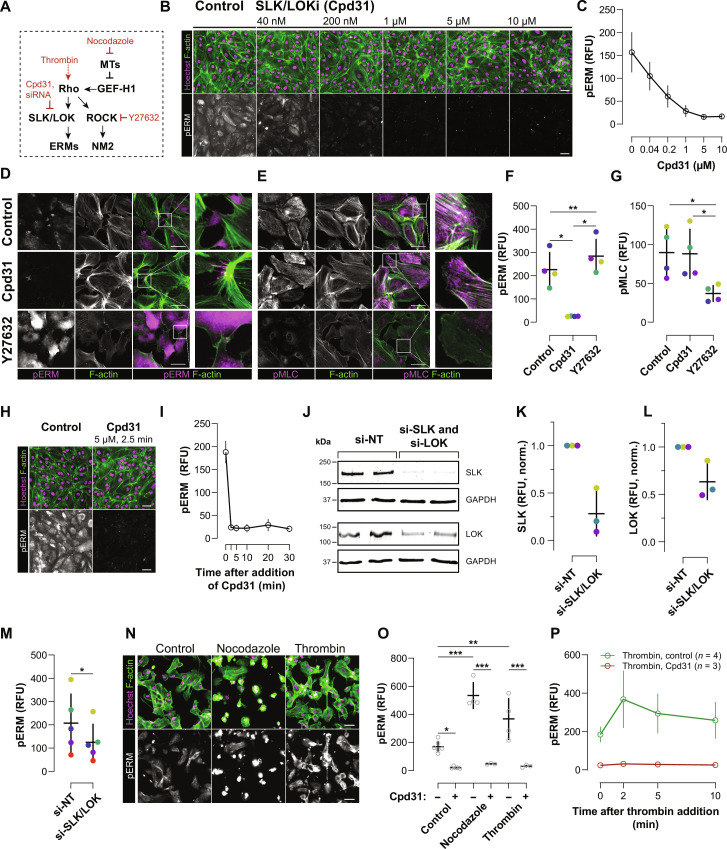
Rho rapidly activates ERMs via SLK/LOK. (**A**) Perturbation strategies to investigate Rho-induced ERM activation. (**B**) pERM signal in HT-HUVEC in response to increasing concentrations of the SLK/LOK inhibitor Cpd31 (4 hours). (**C**) Quantification of (B) using quantitative immunofluorescence (Materials and Methods), means ± SD from *n* = 3 biological replicates. (**D** and **E**) HT-HUVEC treated with Cpd31 (5 μM, 1 hour) or Y27632 (20 μM, 1 hour) and stained for F-actin, pERM, and pMLC. (**F** and **G**) Quantifications of pERM and pMLC in response to Cpd31 and Y27632 using quantitative immunofluorescence. Means ± SD from *n* = 4 biological replicates. Colors of datapoints are matched by replicate. *P < 0.05 and **P < 0.01, one-way ANOVA/Tukey-Kramer. (**H** and **I**) Cells rapidly lose pERM signals when treated with Cpd31 (5 μM). Quantitative immunofluorescence, means ± SD from *n* = 3 biological replicates. (**J**) Lysates of control (si-NT) or SLK/LOK codepleted (si-SLK/si-LOK) cells were analyzed by Western blotting. (**K** and **L**) Quantifications of (J), means ± SD from *n* = 3 biological replicates. Colors of data points are matched by replicate. (**M**) Reduced pERM in cells codepleted of SLK/LOK (si-SLK/LOK), as determined by quantitative immunofluorescence. Means ± SD from *n* = 5 biological replicates. Colors of data points are matched by replicate. *P < 0.05, two-tailed, paired *t* test. (**N**) Treatment with nocodazole (15 μM, 30 min) or thrombin (1 U/ml, 2 min) increased pERM in HT-HUVEC. (**O**) Nocodazole and thrombin-induced increases in pERM were abolished when Cpd31 (5 μM) was present. Means ± SD from *n* = 3 to 6 biological replicates are shown. *P < 0.05, **P < 0.01, and ***P < 0.001, one-way ANOVA/Tukey-Kramer. (**P**) pERM signals rapidly increased upon thrombin stimulation (1 U/ml), except when Cpd31 (5 μM) was present. Means ± SD from *n* = 4 (thrombin) or *n* = 3 (thrombin + Cpd31) biological replicates. Scale bars, 50 μm. RFU, relative fluorescence unit.

To test whether SLK/LOK kinases are involved in ERM activation downstream of Rho in HT-HUVEC (Fig. 6A), we first characterized a recently developed SLK/LOK inhibitor (Cpd31) (*66*). Treatment of HT-HUVEC with Cpd31 caused an almost complete loss of pERM signal, as assessed by quantitative immunofluorescence, with only some detectable pERM signals remaining in peripheral structures resembling retraction fibers (Fig. 6, B and D). pERM signal decreased dose dependently (Fig. 6, B and C) and occurred within 2.5 min of Cpd31 addition at 5 μM (Fig. 6, H and I). No major changes in the actin cytoskeleton of treated cells were apparent (Fig. 6D). Notably, phospho-myosin light chain (pMLC) levels remained unchanged after Cpd31 treatment (Fig. 6, E and G), arguing against ERMs negatively regulating Rho activity in HT-HUVEC. pERM increased upon ROCK inhibition (Fig. 6, D and F), consistent with ERMs being Rho effectors and elevated Rho in the presence of Y27632 (Figs. 4D and 5A). The rapid loss of pERM upon SLK/LOK inhibition is consistent with a high turnover rate of ERM phosphorylation (*36*). Small interfering RNA (siRNA) knockdown experiments confirmed that SLK and LOK mediate ERM phosphorylation in HUVEC (Fig. 6, J to M, and fig. S7, E and F). The incomplete depletion of SLK/LOK by siRNA also resulted in an incomplete loss of pERM (Fig. 6, K to M). We next used acute treatment of cells with thrombin or nocodazole to further assess Rho-dependent changes in pERM signals (Fig. 6A). Thrombin and nocodazole both rapidly activate Rho, thrombin through the G protein–coupled receptor PAR-1 (*67*) and nocodazole through the release of microtubule-sequestered GEF-H1, a Rho-specific GEF, from depolymerizing microtubules (*68*). Both treatments caused rapid, concurrent increases in RhoB sensor activity (Fig. 1E and fig. S8) and pERM signals (Fig. 6, N to P). Strikingly, the increases in pERM signal were potently suppressed when Cpd31 was present (Fig. 6, O and P). Our results therefore demonstrate an essential role of SLK/LOK in rapid ERM activation downstream of Rho and argue for the Rho-SLK/LOK-ERM signaling axis being a highly responsive signaling module for the regulation of actin-membrane attachment and force transmission.

### SLK/LOK-dependent ERM activation regulates cell morphology and is required for Rho-driven cell contractions

To understand how local Rho pulses regulate overall cell contractility and cell shape, we took advantage of the ability to selectively inhibit the Rho downstream targets ROCK/NM2 (using Y27632) and SLK/LOK/ERM (using Cpd31) to identify their individual or combined roles in these processes.

**Fig. 7.**
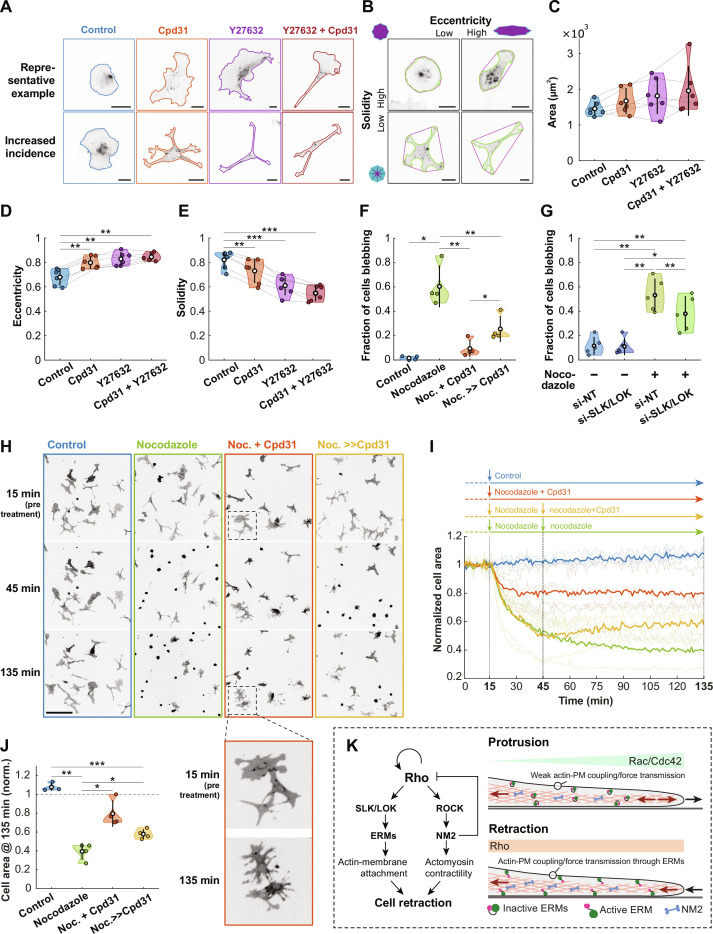
SLK/LOK-dependent ERM activation regulates cell morphology and is required for Rho-driven cell contractions. (**A**) HT-HUVEC plated without (control) or with Cpd31 (5 μM), Y27632 (10 μM), or Cpd31 and Y27632 present. Representative cell shapes with average (top) and increased incidence (bottom) morphologies. (**B**) Examples of varying solidity and eccentricity. (**C**) Average cell area, (**D**) eccentricity, and (**E**) solidity. Means ± SD and data points from *n* = 6 biological replicates (control: 1141 cells, Cpd31: 966 cells, Y27632: 776 cells, and Cpd31 and Y27632: 696 cells). (**F**) Fraction of HT-HUVEC expressing RhoB sensor blebbing after control treatment, 2 hours nocodazole (15 μM), 2 hours nocodazole (15 μM) and Cpd31 (5 μM), or 2 hours nocodazole (15 μM), with Cpd31 (5 μM) added after 30 min [as depicted in schematic in (I), *t* = 135 min]. (**G**) Fraction of blebbing HT-HUVEC, expressing mScarlet3-CAAX and transfected with either si-NT or si-SLK/LOK, control treated or treated with nocodazole (15 μM, 2 hours). Means ± SD and data points from *n* = 5 biological replicates. (**H**) HT-HUVEC expressing RhoB sensor treated as depicted in the schematic in (I). Nocodazole (15 μM) and Cpd31 (5 μM). RhoB sensor localization was used to visualize cells. Zoom-in shows fragmented cells in nocodazole and Cpd31 condition (movie S17). (**I**) Average area covered by cells per FOV as a function of time, normalized to average cell area pre-addition. First treatment 15 min; second treatment, 45 min (black dashed lines). Bold traces denote means of *n* = 4 biological replicates (dashed lines). (**J**) Average cell-covered area per FOV in (I) at *t* = 135 min for each condition, normalized to pretreatment. (I, and J) Means from *n* = 4 biological replicates (control: 14, nocodazole: 13, nocodazole + Cpd31: 14, and nocodazole ≫ Cpd31: 14 FOVs). (**K**) Model for how excitable Rho activity drives cell contractions through both ERM activation via SLK/LOK and ROCK-mediated actomyosin contractility (see main text). *P < 0.05, **P < 0.01, and ***P < 0.001, one-way ANOVA/Tukey-Kramer. Scale bars, 20 μm (A and B) and 200 μm (H).

For cell shape homeostasis, we plated HT-HUVEC without or in the presence of Cpd31, Y27632, or Cpd31 and Y27632 combined; fixed the cells after 2 hours; and stained them with the AF647-conjugated surface label wheat germ agglutinin (WGA647). The advantage of this protocol is that cells have not yet assumed heterogenous cell morphologies before exposure to the inhibitors, facilitating the detection of morphological phenotypes. For all conditions tested, cells attached and spread to similar adhesion areas (Fig. 7C). Y27632 treatment is known to cause elongated cell morphologies and retraction defects (*69*–*71*).We confirmed this and found that inhibition of SLK/LOK had similar effects, with cells having extended cellular processes (Fig. 7A). To quantify drug-induced cell shape changes, we chose eccentricity and solidity as metrics. Eccentricity quantifies cell elongation, and solidity is the ratio of cell area and the area of a cell’s convex hull (cell compactness) (Fig. 7B and Materials and Methods). While the cell spreading area was not affected by either treatment (Fig. 7C), both Cpd31 and Y27632 yielded significantly increased eccentricity and significantly reduced solidity (Fig. 7, D and E), and combined treatment had additive effects on both metrics (Fig. 7, D and E). These results indicated that the Rho effectors ROCK and SLK/LOK both contribute to cell shape homeostasis by restricting extended cellular processes. Cpd31-treated cells remained motile, but often had extended cellular processes and tethered membrane balloons left behind at retraction sites due to impaired membrane cortex attachment (movie S16).

To directly assess the role of Rho-driven ERM activation in membrane retractions, we treated fully adhered cells with nocodazole, as it activates Rho and causes strong retractions, leading to a reduced adhesion surface of cells (fig. S8, A and B). Upon nocodazole addition, cells rapidly contracted and many started blebbing (Fig. 7, F and G, and movie S17). Strikingly, when Cpd31 was added along with nocodazole, both the fraction of cells blebbing and cell contractions were significantly reduced (Fig. 7, F, H, I, and J). Similarly, the fraction of blebbing cells was significantly reduced in nocodazole-treated cells in which SLK/LOK had been depleted by siRNA (Fig. 7G). Together, this demonstrates that SLK/LOK are required for cells to exhibit a contractile response downstream of Rho. In samples where cells were first treated with nocodazole for 30 min to induce Rho-dependent blebbing, the addition of Cpd31 increased cell spreading (Fig. 7, H to J), and a significant fraction of cells recovered from blebbing (Fig. 7F and movie S17). This suggested that the process of bleb retraction not only resolves blebs but also maintains blebbing cells in a blebbing state. Intriguingly, cells treated with combined nocodazole and Cpd31 often had “runaway” protrusions resulting in cell fragments that detached from the main cell body (Fig. 7H, inset). Cpd31 treatment did not prevent nocodazole-induced Rho activation (fig. S8C).

Together, our results demonstrate that the Rho effectors ROCK and SLK/LOK are both critical for Rho-induced cell contractions. Moreover, SLK/LOK activation of ERM downstream of Rho appears to be critical to safeguard cell integrity.

## DISCUSSION

By analyzing the spatiotemporal dynamics of Rho activity in randomly motile HT-HUVEC, we found that Rho was consistently elevated in micrometer and minute-scale cell edge retractions and consistently absent from protrusions. Rho’s elevation in retractions was confirmed using three different FRET probes and a localization-based probe, and is consistent with both recent studies using similar reporters (*19*–*21*) and the generally accepted role of Rho in activating actomyosin contractility through ROCK.

Intriguingly, our analysis revealed that Rho activity in retraction events is excitable, consistent with fast positive feedback and slower negative inhibition. This finding is supported by the multiple instances of excitable Rho activity generating contractile pulses in nonmigratory contexts (*52*, *53*, *57*, *58*).

Analyzing NM2 or the ERM protein ezrin in parallel to Rho dynamics, we found that NM2 accumulated with a minute-scale delay at retracting cell edges relative to Rho activity, whereas ezrin was co-activated without detectable delay. In previous studies on excitable Rho dynamics, actomyosin-associated RhoGAPs have been identified as negative regulators of Rho, in line with our findings that myosin inhibition increased Rho activity (*53*, *57*, *72*). Therefore, it is likely that an actomyosin-associated RhoGAP is recruited to retractions as they evolve, progressively shutting off Rho and ensuring contractions stop.

While positive feedback to Rho most likely involves the recruitment of a RhoGEF (*52*, *57*, *58*, *72*, *73*), it is less clear how this occurs during retractions. The tight spatiotemporal coupling between Rho and ezrin demonstrates that ERMs are early Rho effectors during retractions, and indicates that they could be components of a positive feedback mechanism. Both positive and negative feedback from ERMs to Rho activity have been observed (*34*, *74*, *75*). However, negative feedback through ERMs is unlikely in our case, given the absence of a delay between Rho and ezrin activation and the absence of increased pMLC upon SLK/LOK inhibition. Our results in HT-HUVEC therefore contrast some of the recent findings in polarized epithelial cells, where negative feedback from ezrin to Rho via ARHGAP18 was demonstrated and shown to be required for microvilli formation (*34*, *76*). This suggests cell-type and context-dependent feedback mechanisms regulating Rho. Focal adhesion dynamics may also contribute to regulating Rho during protrusion-retraction cycles. Focal adhesions have been shown to recruit RhoGEFs and RhoGAPs in a maturation-dependent manner, with Rac-specific RhoGEFs associated with newly formed adhesions near the cell edge and Rac-specific RhoGAPs and Rho-specific RhoGEFs with mature adhesion sites deeper in the cell interior (*77*). Furthermore, increased tension on focal adhesions can recruit the Rho-specific RhoGEFs LARG and GEF-H1 (*78*), meaning greater Rho-induced contractility leads to higher Rho activity. Finally, local microtubule disassembly induces retractions (*79*), and the interaction between focal adhesions and microtubules can control Rho activity through the sequestration and release of GEF-H1 (*80*), further supporting a plausible role for GEF-H1 in Rho’s positive feedback.

What are the specific roles of ERMs and NM2 during Rho-dependent cell edge retractions? The initial absence of NM2 in early phases of retractions, despite elevated Rho, can be explained by both the absence of NM2 filaments in branched lamellipodial actin networks and the unavailability of activatable NM2 monomers (*81*, *82*). In contrast, inactive ERMs are partly plasma membrane–localized (*63*, *64*), poised for activation by SLK/LOK kinases to enhance actin membrane attachment. Our results are therefore consistent with a model in which ERMs are the immediate effectors of Rho through SLK/LOK during early phases of retractions to re-establish a link between plasma membrane and peripheral actin networks (Fig. 7K). In adherent cells, these networks typically move radially inwards due to NM2 contractility and/or polymerization-driven treadmilling (*83*, *84*), which in our model drag the membrane inward once Rho-activated ERMs enable force transmission, with ERMs acting as the actin–plasma membrane clutch. Actin network remodeling occurs during early phases of edge retraction, progressively allowing for accumulation of NM2 filaments (*7*, *85*), which in turn exert contractile forces to further accelerate edge retraction. Increasingly remodeled and contractile actomyosin then recruits a putative Rho-specific RhoGAP, which shuts down Rho, with the observed delay, to resolve the retraction.

Rho/ROCK-dependent NM2 activation is important for cell migration and inhibiting ROCK causes a tail-retraction defect (*69*–*71*). Treating cells with the SLK/LOK kinase inhibitor Cpd31 induced similar morphological phenotypes. Although highly motile, these cells had extended cellular processes that often failed to efficiently retract. The model that NM2 and ERMs cooperate during cell contractions was further supported by our results showing that Rho-induced cell contractions were impaired in the presence of the SLK/LOK inhibitor or SLK/LOK depletion by siRNA. Furthermore, cells that were induced to bleb in response to acute Rho activation stopped blebbing and started spreading when SLK/LOK inhibitor was added. Cells treated this way had aberrant “runaway” protrusions that occasionally detached from cells, highlighting the critical importance of Rho-dependent ERM activation through SLK/LOK for cell shape homeostasis and the maintenance of cellular integrity.

In summary, our results show that in endothelial cells, Rho drives membrane retractions through two mechanisms that act sequentially during the cell edge retraction process: SLK/LOK-activated ERMs enhance actin-membrane attachment and force transmission, which enable ROCK-activated NM2 to pull the membrane inward, akin to ERMs acting as a clutch and NM2 as a motor. Previous studies found that initiation of cell protrusions is preceded by a local reduction in membrane-proximal actin or ezrin and that protrusions stalled when actin-membrane attachment was enhanced through synthetic activation of ezrin (*45*, *62*). Therefore, in cells in which the Rho-SLK/LOK-ERM module exists, edge-proximal Rho activity appears to be incompatible with driving membrane protrusions. Because RhoA has been proposed to drive cell edge protrusions by activating mDia1 (*13*), it will be important to investigate whether edge-proximal RhoA and ERMs are spatiotemporally correlated in these contexts.

Our findings raise several important questions to be addressed in future research. Which are the RhoGEFs and RhoGAPs that mediate Rho excitability in a cell type and context-dependent manner, and is Rho excitability a general phenomenon in motile cells? We found that Rho activity is self-limiting in randomly motile cells, with activated NM2 being part of a slow negative feedback mechanism. If Rho activity is excitable during directed cell migration as well, then this implies that Rho activity is pulsatile rather than forming a stable rear-front gradient. Beyond cell motility and given accumulating evidence for actin-membrane attachment in regulating diverse cell morphogenetic processes, including membrane tension propagation (*86*), we anticipate that our identification of ERMs as highly responsive Rho effectors will have far-reaching implications for our understanding of cell morphogenesis and migration.

## MATERIALS AND METHODS

### Cell culture

HT-HUVEC, generated by stable transduction of primary HUVEC from mixed (male, female) donors with hTERT have been described (*29*). They were cultured in either endothelial cell growth medium 2 (Lonza, CC-3162), (PromoCell, C-22011), or in EndoGRO VEGF (MilliporeSigma, SCME002), supplemented with hygromycin (50 μg/ml). HT-HUVEC stably expressing reporter constructs were generated by lentiviral transduction, followed by antibiotic selection [blasticidin (10 μg/ml), G418 (0.5 mg/ml), or puromycin (0.5 µg/ml)]. Cells expressing multiple fluorescence-based reporters were generated by sequential lentiviral transductions and antibiotics selections. Stably transduced cells were maintained in the presence of blasticidin, G418, puromycin as applicable. Human embryonic kidney (HEK) 293FT cells (not authenticated), used for lentivirus production, were grown in Dulbecco’s modified Eagle medium (Thermo Fisher Scientific, 11965092) supplemented with 10% fetal bovine serum (FBS) (Corning, 35-077-CV) and 5% GlutaMAX (Thermo Fisher Scientific, 35050061). All cells were grown in the absence of antimicrobial agents, and the absence of mycoplasma in cell cultures was routinely verified using a polymerase chain reaction (PCR) test (*87*). A complete list of cell lines used with references to figure panels is shown in table S1.

### Antibodies and reagents

Rabbit anti-phospho ezrin(T567)/radixin(T564)/moesin(T558) (3726S; used at 1:400), rabbit anti-phospho MLC2 Ser19 (3671S; used at 1:400), and rabbit anti-SLK (41255S; used at 1:1000) were purchased from New England Biolabs; rabbit anti-LOK (A300-399A; used at 1:5,000) was from Bethyl Laboratories; mouse anti–glyceraldehyde-3-phosphate dehydrogenase (GAPDH) (MA5-15738; used at 1:2,000) was from Thermo Fisher Scientific; basic fibroblast growth factor (bFGF) was from Cedarlane Labs (CL104-02-50UG); thrombin was from MilliporeSigma (T4648-1KU); ML7 was from Enzo (BML-EI197-0010); Y27632 was from Cedarlane Labs (Y1000-1MG); rapamycin was from LC Laboratories (R-5000); nocodazole was from Cedarlane Labs (13857-5); and SLK/LOK inhibitor (*66*) “Cpd31” was from MedChemExpress (HY-132868). Phalloidin conjugated to Alexa Fluor 488 (AF488) was from New England Biolabs (8878S), Hoechst 33342 was from Thermo Fisher Scientific (H3570), AF-conjugated secondary antibodies was from Thermo Fisher Scientific, IRDye800CW and IRDye680RD-conjugated secondary antibodies were from LI-COR Biosciences, wheat germ agglutinin conjugated to AF647 (WGA-AF647) was from Thermo Fisher Scientific (W32466), bovine collagen type I was from Advanced Biomatrix (5005-100ML), paraformaldehyde solution was from Thermo Fisher Scientific (50-980-487), bovine serum albumin (BSA) from BioShop Canada (ALB001.250), and hygromycin, blasticidin, puromycin, and G418 were from Invivogen (ant-hg-1, ant-bl-1, ant-pr-1, and ant-gn-1, respectively). The following siRNAs were purchased from the Horizon Discovery: si-NT (on-TARGETplus, nontargeting pool, D-001810-10), si-KIF11 (on-TARGETplus, L-003317-00), si-SLK (siGENOME, M-004168-01), and si-LOK (siGENOME, M-004168-01).

### DNA constructs

mCherry-FKBP-GEF(TIAM1) (Addgene, 85156), mCherry-FKBP-GEF(ARHGEF1) (Addgene, 85152), and Lyn_11_-FRB (Addgene, 155228), all in pCAGEN, have been described previously (*29*, *45*). mCherry-FKBP-GEF(ITSN1), in pCAGEN, included mCherry, FKBP, and the DH domain of human ITSN1 (amino acids 1218 to 1429), which was PCR-amplified from a human ORFeome clone (V5.1, clone 56297).

mCherry-FKBP-GAP(ARHGAP29), in pCAGEN, included mCherry, FKBP, and the GAP domain of human ARHGAP29 (amino acids 668 to 900), which was PCR-amplified form HT-HUVEC cDNA. pLV-RaichuEV-Rac-IRES-Blast and pLV-RaichuEV-Cdc42-IRES include a codon-diversified YPet to reduce similarity with mTurquoise at the nucleotide level to enable their lentiviral transduction. Both reporters and constructs have been described previously (*43*–*45*).

To generate pLV-RhoA2G-IRES-Blast, RhoA2G, provided by O. Pertz (University of Bern, Switzerland) (*37*) was PCR-amplified and inserted into pLV-EF1a-MCS-IRES-Blast (*29*)(Addgene, 85133) using Gibson assembly (*88*).

DORA-RhoA, a dimerization optimized reporter of activation for RhoA (*38*, *39*), with codon-diversified dCer3 and L9Hx3 linker, and a sensor-dead version of DORA-RhoA (PKN-L59Q) were provided by Y. Wu (University of Connecticut, USA). To generate pLV-DORA-RhoA-IRES-Blast, DORA-RhoA was PCR-amplified and inserted into pLV-EF1a-MCS-IRES-Blast using Gibson assembly.

The RhoB sensor used in this study has been described previously (*18*). Here, pLV-RhoB sensor-IRES-Blast was generated through PCR amplification of RhoB from HT-HUVEC cDNA and DORA-RhoA minus RhoA from pLV-DORA-RhoA-IRES-Blast, and both PCR products were inserted into pLV-EF1a-MCS-IRES-Blast using Gibson assembly. A sensor-dead version of the RhoB sensor was generated similarly by using DORA-RhoA-PKN(L59Q) as a PCR template for the DORA-RhoA minus RhoA sequence.

pLV-mRuby3-MLC-IRES-Neo was created by PCR-amplifying MLC (MYL9) and mRuby3 using pLV-Ftractin-mRuby3-p2A-mTurquoise-MLC-IRES-Blast as template (*29*)(Addgene, 85146) and by inserting the products into BamHI/NotI–digested pLV-EF1a-MCS-IRES-Neo (*29*) (Addgene, 85139) using Gibson assembly.

The stoichiometric ezrin-mRuby3/miRFP680 reporter, in pLV-EF1a-MCS-IRES-Neo, consisted of miRFP680-NES (*89*), followed by a p2A ribosomal skipping sequence (*90*) and full-length ezrin (PCR-amplified from HT-HUVEC cDNA), C-terminally tagged with mRuby3. The sequence encoding miRFP680 was downloaded from Addgene (136557, pmiRFP680-N1), optimized for mammalian expression using a web-based codon-optimization tool (Thermo Fisher Scientific), and synthesized (gBlock, IDT DNA) with a nuclear export signal (LALKLAGLDI) (*91*) and p2A sequence appended. A nonphosphorylatable version of this reporter was created analogously by introducing a T567A mutation in ezrin through PCR.

The localization-based Rho activity reporter dTomato-2xrGBD (rhotekin G protein binding domain) has been described (Addgene, 129625) (*19*). To make it compatible with lentiviral transduction, one of the two rGBDs was codon-diversified and synthesized (gBlock, IDT DNA). To enable correction for cell geometry/localization bias, dTomato-2xrGBD was coexpressed with an miRFP680-NES (see above) through Gibson assembly of miRFP680-NES-p2A and dTomato-2xrGBD into BamHI/NotI–digested pLV-EF1A-MCS-IRES-Neo.

A construct for the expression of cytoplasmic mRuby3 was generated by inserting mRuby3 into BamHI/NotI–digested pLV-EF1a-MCS-IRES-Neo using Gibson assembly.

The live-cell membrane marker mScarlet3-CAAX, in pLV-EF1a-MCS-IRES-Puro (*29*)(Addgene, 85132), was created by PCR amplification of mScarlet3 from pmScarlet3-Giantin_C1 (Addgene, 189773) (*92*) and Gibson assembly into a digested pLV-EF1a-MCS-IRES-Puro vector that already contained the KRas4B CAAX motif. Plasmid constructs generated during this study can be obtained from Addgene (www.addgene.org/Arnold_Hayer/).

### Lentivirus production

Lentivirus was generated in HEK293FT cells as described (*29*). Briefly, confluent 10-cm dishes of cells were cotransfected with a transfer vector containing the gene of interest (15 μg) and the third-generation packaging plasmids pMDLg/pRRE (5 μg), pRSV-rev (5 μg), and pCMV-VSVG (5 μg) using Lipofectamine 2000 (50 μl; Thermo Fisher Scientific, 11668027), in final 10 ml of OptiMEM (Thermo Fisher Scientific, 31985070). Viral supernatants were collected at 48 hours after transfection, 0.22 μm–filtered (VWR CA28143-310), concentrated using centrifugal filter units (100-kDa cutoff; MilliporeSigma, UFC910024), aliquoted, and either used immediately or stored at −80°C.

### Cell plating, live-cell, and fixed-cell microscopy

Optical 96-well glass-bottom plates (Cellvis P96-1.5H-N) were coated with bovine collagen-type I, 31 μg/ml in phosphate-buffered saline (PBS), for 2 to 4 hours at 37°C. Cells were plated at densities and for durations before fixation or live-cell imaging as specified. For live-cell imaging, cells were overlaid with a CO_2_-independent live-cell imaging solution (LIS) with low autofluorescence, composed of 125 mM NaCl, 5 mM KCl, 1.5 mM MgCl_2_, 1.5 mM CaCl_2_, 10 mM d-glucose, 20 mM HEPES (pH 7.4), 1% FBS, and bFGF (5 ng/ml), and plates were sealed during imaging using aluminum microplate seals (PolarSeal, Thomas Scientific, 1152A34). Cells were fixed by adding fixation solution (4% formaldehyde in PBS) at a ratio of 1:1 to culture medium or LIS (final 2% formaldehyde) and incubated for 15 min at room temperature (RT). Following two PBS washes, cells were either stained with WGA-AF647 (2.5 μg/ml in PBS, 10 min at RT), or permeabilized for immunofluorescence staining using ASBB, a permeabilization/ blocking solution (10% FBS, 1% BSA, 0.1% Triton X-100, 0.01% NaN_3_, in PBS) for 30 min. Incubation with primary antibodies, diluted in ASBB, was done either at RT for 2 h or at 4°C overnight. Incubation with secondary antibodies, diluted 1:1000 in ASBB, was done at RT for 1 h. Fixed cells were imaged overlaid with PBS.

Imaging data were acquired using one of the four live-cell imaging systems described below, as indicated in Table 1:

**Table 1.** Live-cell imaging systems used for each figure/movie.

Live-cell imaging system #	Used for figure/movie
1	Fig. 1F, H to K
2	Fig. 1D; 2C; 3A to D; Fig. 3I, J: Fig. 4B; fig S3; fig S5; movie S8 to S10
3	Fig. 7H, fig. S7, movie S17
4	Fig. 1A to E; Fig. 2; Fig. 3 E to H; Fig. 4 to 6; Fig. 7A to G, I to J; fig S1; fig. S2; fig S4; fig. S6; fig. S8; movie S1 to S7; movies S11 to S16

**Live-cell imaging system 1.** Fully automated widefield/Yokogawa spinning disc confocal fluorescence microscope system (Intelligent Imaging Innovations, 3i), built around a Nikon Ti-E stand, equipped with Nikon’s Perfect Focus System, a 40× 1.3 numerical aperture (NA) Plan Fluor oil objective, a 3i laser stack (405, 442, 488, 514, 561, and 640 nm), a broad-range fluorescence light source with integrated excitation filter wheel, (Lambda XL, Sutter), a Yokogawa CSU-W1 scanning head with dual camera port and emission filter wheels, two scientific complementary metal-oxide semiconductor (sCMOS) cameras (Andor Zyla 4.2); enclosed by an environmental chamber (Haison); and controlled by SlideBook software (3i).

**Live-cell imaging system 2.** Fully automated fluorescence microscope system (ImageXpress Micro XL, Molecular Devices), equipped with white light light-emitting diode (LED) light source (SOLA, Lumencor), a Zyla 5.5 sCMOS camera (Andor), and a 20× 0.75 NA Plan Apo air objective (Nikon) and controlled by MetaXpress software.

**Live-cell imaging system 3.** Fully automated widefield fluorescence microscope system (Nikon), built around a Nikon TI-E stand, equipped with Nikon’s Perfect Focus System, a 10× 0.45 NA Plan Apo air, a 20× 0.75 NA Plan Apo air, a 40× 1.3 NA Plan Fluor oil immersion objective, a liquid light guide-coupled white-light LED light source (SOLA SE UV-nIR, Lumencor), excitation and emission filter wheels (Lambda 10-3, Sutter), and a sCMOS camera (Prime-BSI, Photometrics); enclosed by a custom-built environmental chamber (Digital Pixel); and controlled using Nikon NIS Elements AR software.

**Live-cell imaging system 4.** Fully automated widefield fluorescence microscope system (Nikon), built around a Nikon TI2-E stand, equipped with Nikon’s Perfect Focus System, a 10× 0.45 NA Plan Apo air, a 20× 0.75 NA Plan Apo air, a 40× 1.3 NA Plan Fluor oil immersion objective (Nikon), a liquid light guide-coupled multispectral LED light source (SpectraX, Lumencor), a dual-camera image splitter (TwinCam, Cairn) with a custom-integrated high-speed emission filter wheel (HS-1025, FLI), two sCMOS cameras (Orca Fusion-BT, Hamamatsu), and a triggered device hub (NI-BB, Nikon); enclosed by a custom-built environmental chamber (Digital Pixel); and controlled using Nikon NIS Elements AR software.

### Transient transfection of cDNA, synthetic Rho, Rac, and Cdc42 (in)activation using rapamycin-induced heterodimerization

HT-HUVEC expressing RhoB sensor, RaichuEV-Rac, or RaichuEV-Cdc42 was plated in collagen-coated wells of 96-well glass-bottom plates at 15,000 cells per well the day before the transfection. The day of the transfection, the culture medium was replaced with antibiotic-free full growth medium, 80 μl per well. Then, 0.2 μg of DNA (LynFRB:mCherry-FKBP constructs, 5:1 w/w) and 0.25 μl of Lipofectamine 2000 (Thermo Fisher Scientific, 11668019), diluted in 20 μl OptiMEM (Thermo Fisher Scientific, 31985070), was added as per the manufacturer’s recommendation. The transfection mix was replaced after 2 hours with full growth medium. Sixteen to 18 hours later, cells were overlaid with LIS, and images were captured using live-cell imaging system 1 using a 40× 1.3 NA oil objective lens at 0.33 μm per pixel resolution (2 × 2 binning). FRET, CFP, and mCherry channels were acquired sequentially. Sixty images were captured at 15-s intervals. Rapamycin was added after time-point 10 at 0.5 μM final concentration.

### Image analysis - background subtraction, cell segmentation, and cell tracking

Background images for each channel were generated by imaging wells without cells and filled with LIS. Multiple images from multiple wells were averaged and subjected to circular image filtering using MATLAB functions imfilter and fspecial. To adjust raw images of cells, the median intensity of neighboring pixels from the average background image was subtracted. Cell masks were generated using histogram-based thresholding based on pixel intensity distribution of the yellow fluorescent protein (YFP)–FRET channel. To smooth the cell edge and refine the mask, Gaussian circular averaging filters were applied. To remove small debris trails attached to/touching cells, MATLAB functions imopen and strel were used to remove edge elements with a radius less than 1 pixel. Subsequent cell trajectories were created using a nearest-neighbor consecutive pairing algorithm as described previously (*17*).

### FRET and CFP channel alignment, FRET/CFP ratio calculation, and fluorophore bleaching correction

Methods for FRET and CFP channel registration have been described previously (*17*). FRET/CFP ratios were computed as per-pixel ratios from background-subtracted, registered, and noise-filtered FRET and CFP images. By plotting FRET/CFP averaged per field of view over time from untreated samples, a bleaching correction curve was generated, displaying exponential-like decay. Assuming that FRET/CFP across multiple untreated cells remained constant throughout an experiment, the FRET ratio array for each time frame was divided against the curve, normalizing it.

### Mapping cell-edge velocity and RhoGTPase activities

Cell edge velocities and RhoGTPase activities in peripheral coordinate windows were analyzed as described previously (*17*). Briefly, the cell edge of segmented cells was divided into 900 equally spaced coordinate windows. For each coordinate window, velocity vectors were formed by the dot product between a unit vector normal to the cell edge and the displacement vector from frame to frame. Coordinate windows were then binned into groups of five, forming 180 averaged coordinate windows, each with their own velocity vector. Coordinate windows had an adjustable depth parameter, including 0.98, 2.0, 3.3, 4.9, 6.5, and 8.1 μm. FRET/CFP or any expressed protein’s value for each coordinate window was the average value of all pixels assigned to the window. To remove stochastic noise from edge velocity and FRET activity data, the MATLAB function ndnanfilter was applied.

### Thresholding of protrusions and retractions

Cell edge velocity maps were first thresholded either above 3.84 μm/min, creating a map of only protrusive activity, or below −3.84 μm/min to depict retractive activity. Using MATLAB’s bwareaopen function, we then applied a minimum size threshold of 25 pixels to each map, leaving distinct protrusion and retraction events above the size threshold. For each event, data such as area size, pixel IDs, time range, cell edge coordinate range, average edge velocity, average FRET/CFP were recorded.

### Cross-correlation analysis

All cross-correlation analyses were performed using MATLAB’s xcorr function. Cell edge velocity and protein activation arrays were shifted to a standard normal ~ (0,1) distribution using the central limit theorem. To avoid a built-in time lag when comparing FRET/CFP to edge velocity at a given frame *x*, edge velocities from frame *x* − 1 ➔ *x* and *x* ➔ *x* + 1 were averaged. Then, cross-correlation was computed for each window coordinate vector of all time points, yielding 180 correlation vectors. These were averaged to form one cross-correlation vector per cell. The overall cross correlation for a given experimental condition was obtained by averaging the vectors from all cells analyzed.

### Edge velocity versus RhoGTPase activity buildup plots

Previously generated edge velocity accompanying FRET/CFP arrays from sample cells were loaded and processed using MATLAB. To avoid bias, only edge velocity maps were visualized when choosing protrusion-retraction transition events. Rectangular regions of interest undergoing protrusion-retraction cycling were identified. A region of interest spanning 15 coordinate windows or 8.3% of the cell perimeter was created. Only events with >20 frames or 8.3 min of averaged positive edge velocity preceding the transition event were used. The exact transition point from protrusion to retraction was identified as the first frame *x* with (i) negative acceleration, (ii) positive edge velocity at frame *x* − 1, and negative edge velocity at frame *x*. Between frame *x* − 1 and *x*, the frame with lowest absolute-valued edge velocity was used as the transition point. The length of the rectangular region of interest (ROI) was 41 frames using ±20 frames before and after the transition frame (fig. S3, A and B). Edge velocity and FRET/CFP at a chosen edge depth were plotted on the same graph (fig. S3C).

### Ezrin-mRuby3/miRFP680 ratio calculation

Ezrin-mRuby3 and miRFP680 image channels were background subtracted and segmented using the FRET channel–derived cell mask. Circular averaging image filters were applied to each channel (ndanfilter and fspecial in MATLAB). Each channel was normalized to its bleaching curve. Then, the per-pixel ezrin-mRuby3/miRFP680 ratio was computed. The 1st and 99th percentile values of the ratio were calculated and used as the lower and upper limits, i.e., any ratio value less than the first percentile was rounded up to the first percentile, and any values above the 99th percentile were rounded down to the 99th percentile. This was to avoid outlier extreme ratio values due to stochastic noise.

### Ezrin-WT/T567A-mRuby3/miRFP680 ratios in pERM hotspots

HT-HUVEC stably expressing either ezrin-WT-mRuby3-p2A-miRFP680 or ezrin-T567A-mRuby3-p2A-miRFP680, seeded at 1000 cells per well in collagen-coated 96-well glass-bottom plates, were treated with 5 μM nocodazole for 1 hour to increase Rho activity dynamics. Cells were then fixed, stained with anti-pERM antibodies, and imaged using a 40× 1.3 NA oil immersion objective. Cells were chosen and manually cropped. For each cell, areas with elevated pERM were identified by thresholding pERM-AF488 signals at 8× over background. Within the resulting mask mean pERM intensity and mean ezrin-WT/T567A-mRuby3/miRFP680 ratios were calculated. The resulting values were normalized to the respective whole-cell mean values and reported for individual cells.

### Quantitative immunofluorescence

Fixed cells in 96-well glass bottom dishes were stained with 1:400 diluted anti-pERM or anti-pMLC antibody, 1:1000 diluted AF568-conugated secondary antibody, 1:200 diluted AF488-conjugated phalloidin, and 1:10,000 diluted Hoechst. Sixteen images were captured per well using a 20× 0.75 NA air objective at 0.33 μm per pixel resolution. Images with visible staining or imaging artifacts were discarded, and the remaining analysis was fully automated. Cell nuclei were detected on the basis of the Hoechst images using a previously described MATLAB routine (*93*). For quantification of pERM signals, the nuclei in the resulting mask were dilated by 7 pixels (2.3 μm) and the resulting image regions used to determine per-cell pERM signals using background-subtracted pERM images. For quantification of pMLC signals, cell-covered areas were segmented using phalloidin-AF488 signals, and pMLC signals were quantified in the resulting mask.

### RhoGTPase activity response to rapamycin-induced Rho, Rac, and Cdc42 (in)activation

Images were processed, and raw FRET/CFP ratios were computed as described above. An interactive custom MATLAB routine was then used to manually identify cells coexpressing mCherry-FKBP–tagged RhoGTPase regulatory domains and Lyn-FRB based on the presence of mCherry fluorescence. ROIs were drawn within cells identified this way, and the corresponding raw FRET/CFP time courses were automatically computed. For controls, ROIs were drawn in cells without mCherry expression. All time courses were first individually normalized to the average FRET/CFP of the time points before rapamycin addition. Then, the time courses of mCherry-expressing cells were normalized by averaged control time courses, before averaging per condition, across two biological replicates.

### RhoGTPase activity responses to drug additions

Cells were plated 3 to 4 hours before imaging in collagen-coated glass-bottom 96-well plates at a density of 2000 cells per well. Full growth medium was replaced with LIS 1 hour before imaging. Time-lapse sequences were acquired using a 20 × 0.75 NA air objective at 0.65 μm per pixel resolution and at 1-min intervals for 60 min. Drugs diluted in LIS and LIS only (control) were added to wells after indicated times. Image data were processed as described above, and a pixel-wise FRET/CFP calculation was performed. For each field of view, average Rho-FRET activity per frame was calculated and normalized (i) to its own FRET average in the frames before drug addition, and (ii) to the mean control FRET response from all control FOVs. These normalizations scaled each FRET response to one during the before drug addition period and accounted for any increase in FRET activity due to the addition of control LIS.

### Cell shape analysis

HT-HUVEC were seeded at 750 cells per well on collagen-coated 96-well glass-bottom plates in either full growth medium (control) or full growth medium supplemented with either Cpd31 (5 μM), Y27632 (10 μM), or both Cpd31 (5 μM) and Y27632 (10 μM). Cells were incubated for 2 hours at 37°C before fixation with 2% formaldehyde in PBS and staining with AF647-conjugated WGA (2.5 μg/ml in PBS, 10 min at RT) as a membrane marker and Hoechst (1:10,000) to stain the nuclei. Images were then acquired using live-cell imaging system 4, acquiring 16 sites per well across 30 to 40 wells at ×20 magnification with a pixel size of 0.33 μm. Image processing was performed using custom MATLAB code. Objects (cells) were automatically detected and cropped from each image. To exclude cell debris and other particles, only objects with an area greater than 3000 pixels were retained. Object properties (centroid coordinates, area, eccentricity, solidity, major axis length, minor axis length, perimeter, major axis orientation, and mean intensity) were then extracted from each cropped image, as defined by MATLAB’s built-in regionprops function. Solidity is defined as the ratio of the area occupied by the cell over the area of the convex hull of the cell shape. Eccentricity is defined as the ratio between the interfocal distance and the major axis of an ellipsoid. The number of objects in the nuclear and cell masks was used to discard objects without a nucleus (i.e., large cell debris) and cases with more than one nucleus but only one object (i.e., cells touching each other and binucleate cells).

### Cell area and blebbing quantifications in drug-treated cells

RhoB sensor-expressing HT-HUVEC were seeded at 1000 cells per well in collagen-coated 96-well glass-bottom plates and incubated in full growth medium for 3 to 4 hours before replacing the full growth medium by LIS. At specified times during image acquisition, LIS (control), nocodazole (15 μM), both nocodazole (15 μM) and Cpd31 (5 μM), or Cpd31 (5 μM) was administered to the corresponding wells. Image series were acquired using live-cell imaging system 3 or 4 at 1-min intervals over 135 min in the YFP channel using ×10 magnification and 0.65 μm per pixel resolution. Image analysis was performed using custom MATLAB code. The image series were registered using the hardware stage position data, and the background illumination profile was corrected using images from wells left without cells. Cell segmentation was then performed using a local minima histogram-based segmentation algorithm to extract cell occupancy area per field of view per frame. Then, cells were manually counted and classified as either blebbing or spread from images taken from one frame before drug addition and at the end of the time series.

### siRNA knockdown of SLK/LOK, Western blot analysis, blebbing, and pERM quantification in siRNA-treated cells

Before siRNA transfection, HT-HUVEC were trypsinized, pelleted, and resuspended in OptiMEM media (Thermo Fisher Scientific, 31985070). siRNAs were transfected at 20 nM (for SLK/LOK codepletion, 10 nM si-SLK and 10 nM si-LOK) using Lipofectamine RNAiMAX (Thermo Fisher Scientific, 13778100) in OptiMEM, and following the manufacturer’s “reverse transfection” protocol. A nontargeting siRNA pool (si-NT) was used as negative control and si-KIF11 as a positive control for transfection. For Western blot analysis, 250,000 HT-HUVEC were transfected per condition using 7.5 μl of RNAiMAX in 2-ml final volume and plated into wells of a collagen-coated six-well plate. For imaging/blebbing quantification, per condition, 10,000 HT-HUVEC stably expressing mScarlet3-CAAX were transfected using 0.25 μl of RNAiMAX in 100-μl final volume and plated in wells of a collagen-coated glass-bottom 96-well plate. The transfection mix was replaced by full growth medium after 6 to 8 hours, and cells were analyzed after 72 hours.

For Western blot analysis, cells were washed using PBS and then lysed in 200 μl of ice-cold lysis buffer [25 mM HEPES (pH 7.4), 100 mM NaCl, 5 mM EDTA, and 1% Triton X-100 supplemented with “cOmplete” protease inhibitors (MilliporeSigma, 11836170001)]. Postnuclear supernatants were prepared, and lysates equivalent of 15 μg protein per lane were resolved by SDS–polyacrylamide gel electrophoresis, followed by transfer to polyvinylidene difluoride membranes, which were probed using anti-SLK (1:1000), anti-LOK (1:5000), and anti-GAPDH (1:2000) antibodies at 4°C overnight. Bands were visualized using anti–mouse-IRDye680RD, anti–rabbit-IRDye800CW (both at 1:20,000), and an Odyssey CLx imager (LI-COR). Image Studio (LI-COR) was used to quantify the bands.

For imaging/blebbing quantification, cells were trypsinized and replated at 72 hours after transfection, into sixwells per condition, at 1000 cells per well, using the same collagen-coated 96-well glass-bottom plate. Three hours later, cells were either control treated (1:10,000; in growth medium; 5 mM HEPES (pH 7.4) and Hoechst] or treated with 15 μM nocodazole [1:10,000; in growth medium; 5 mM HEPES (pH 7.4) and Hoechst] for 2 hours. A single time point of live cells was captured using a 10 × 0.45 NA air objective. For the relevant conditions, the fraction of blebbing/total cells was determined by manual classification and counting of cells in acquired images. To assess the effect of si-SLK/LOK on pERM, a similar protocol was followed, except that cells were fixed without replating, followed by quantitative immunofluorescence analysis.

### Statistical testing and reproducibility

For comparison of two groups, the nonparametric Mann-Whitney *U* rank sum test or a two-tailed paired *t* test was used. For comparison of multiple groups, a one-way analysis of variance (ANOVA) followed by Tukey-Kramer’s pairwise comparisons was performed. All data shown are from multiple biological and technical replicates, as specified in the figure legends. Statistical analyses were performed using MATLAB or GraphPad Prism.
