# Supplementary material for: Excitable Rho dynamics control cell shape and motility by sequentially activating ERM proteins and actomyosin contractility
Source: Sci Adv. 2024 Sep 6;10(36):eadn6858. doi: 10.1126/sciadv.adn6858 (PMC11378911; doi:10.1126/sciadv.adn6858)
Supplement: Supplementary file 1 — Figs. S1 to S8 Legends for movies S1 to S17 Legend for data S1 [file sciadv.adn6858_sm.pdf]

Science Advances
AAAS

Supplementary Materials for

Excitable Rho dynamics control cell shape and motility by sequentially activating ERM proteins and actomyosin contractility

Seph Marshall-Burghardt *et al.*

Corresponding author: Arnold Hayer, arnold.hayer@mcgill.ca

Sci. Adv. **10**, eadn6858 (2024)
DOI: 10.1126/sciadv.adn6858

The PDF file includes:

Figs. S1 to S8
Legends for movies S1 to S17
Legend for data S1

Other Supplementary Material for this manuscript includes the following:

Movies S1 to S17
Data S1

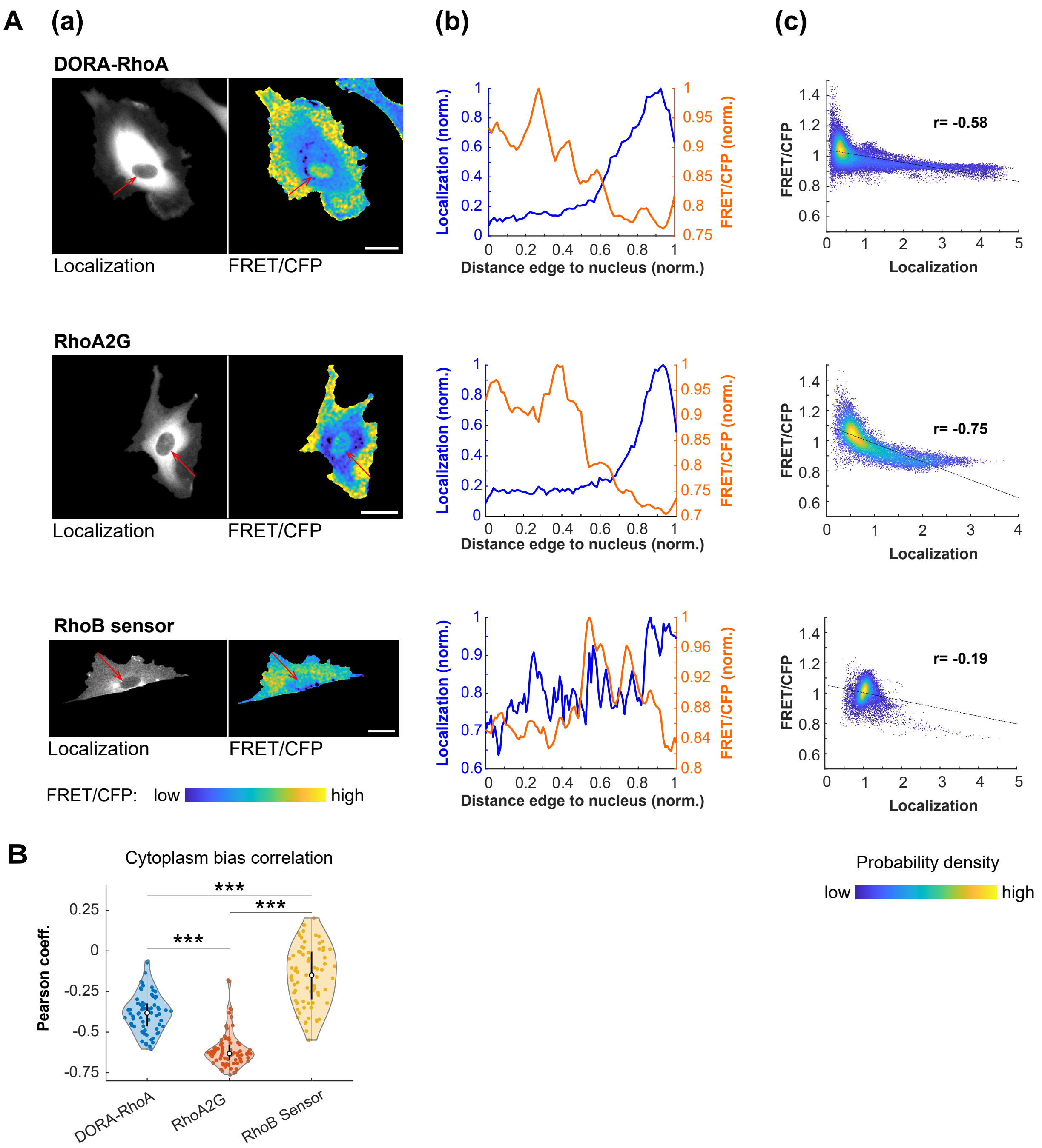

Figure S1. Localization/cell geometry-imposed bias on activity reported by the Rho FRET probes DORA-RhoA, RhoA2G, and RhoB sensor

(A) (a) Side-by-side comparisons of FRET probe localizations (left) and FRET/CFP ratios (right) for DORA-RhoA, RhoA2G, and RhoB sensor. Scale bars, 25µm. Quantifications along the red arrows, manually drawn from the cell edge to the nucleus, are shown in **(b)**. **(b)** Line plots from the images in **(a)** depicting normalized localization compared to normalized FRET/CFP ratio. Both were normalized to the maximum value along the line profiles. **(c)** Per-pixel correlation between localization intensity and FRET/CFP ratio of the two images in **(a)**, with FRET/CFP plotted as a function of normalized localization signal. Pearson R correlation coefficients were calculated using MATLAB corrcoeff function, and line of best fit was plotted using MATLAB polyfit function. **(B)** Compiled Pearson R correlation coefficients for DORA-RhoA, RhoA2G, and RhoB sensor. Individual frames from time-lapse sequences from biological replicates were chosen at random and the correlation coefficient between normalized localization and FRET/CFP were calculated. DORA-RhoA: n= 69 frames from 23 cells, 2 independent trials. RhoA2G: n=75 frames from 25 cells, 2 biological replicates. RhoB sensor: n=75 frames from 25 cells, 3 biological replicates. The bolded circle in violin plot shows dataset median, and bolded black lines show 25th and 75th percentiles. ***$p<0.001$, one-way ANOVA/Tukey-Kramer.

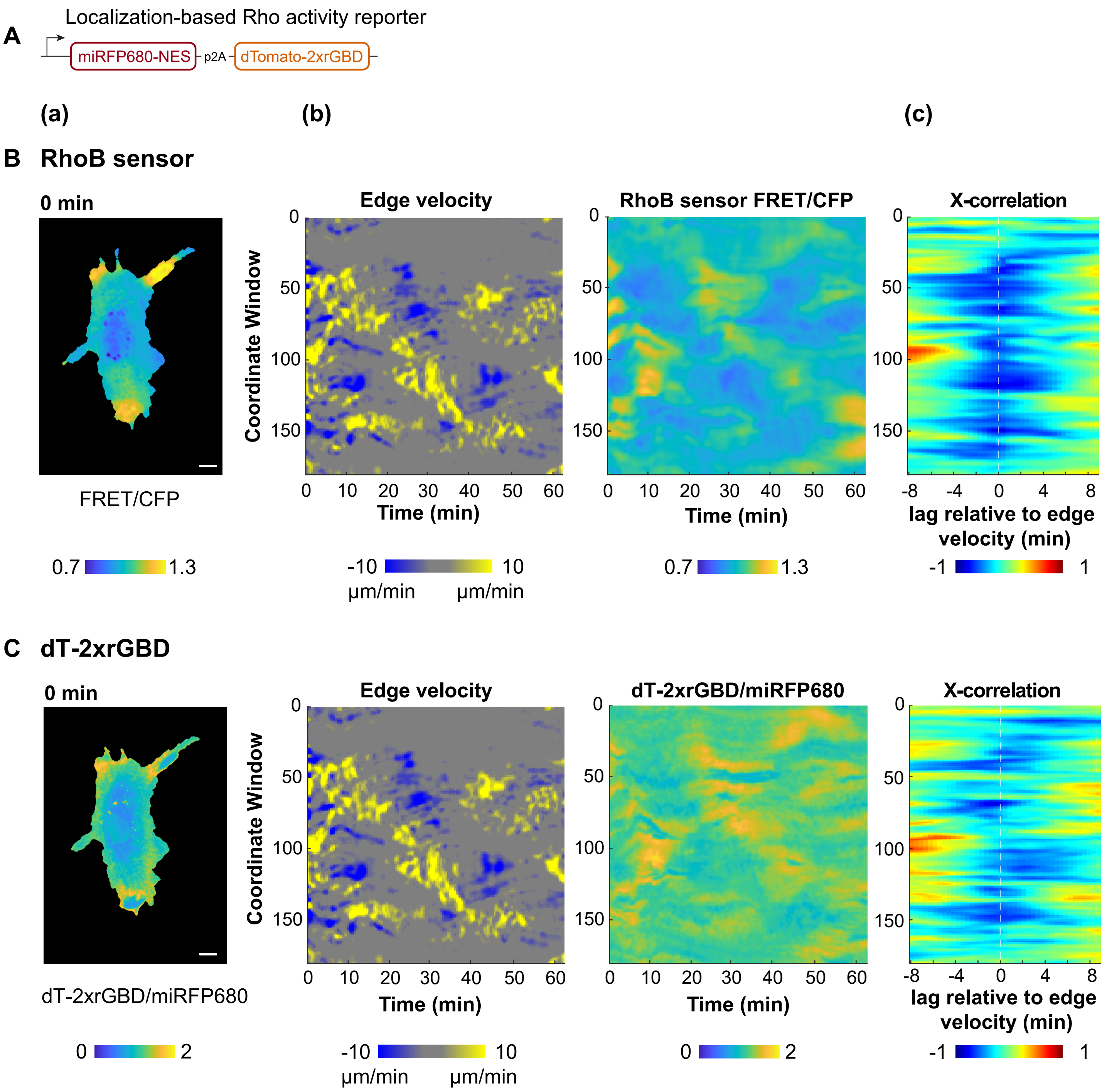

Figure S2. Spatiotemporal analysis of RhoB sensor and the localization-based Rho activity reporter dTomato-2xrGBD in the same cell

(A) Schematic of localization-based Rho activity reporter, consisting of dTomato-2xrGBD and cytoplasmic miRFP680-NES. miRFP680-NES was co-expressed for normalization to cell geometry. **(B)** RhoB sensor. **(C)** dTomato-2xrGBD. **(a)** Rho activity as determined by RhoB sensor FRET/CFP and dTomato-2xrGBD/miRFP680 ratios. **(b)** (Left) Edge velocity maps and (right) spatiotemporal activity maps of cell in **(a),** measured within 1.95µm from the cell edge, from 62.5min time-lapse acquisitions. The same edge velocity maps are shown in **(B)** and **(C)** to facilitate comparison with spatiotemporal activity maps. **(c)** Cross-correlation between edge velocity and spatiotemporal activity maps.

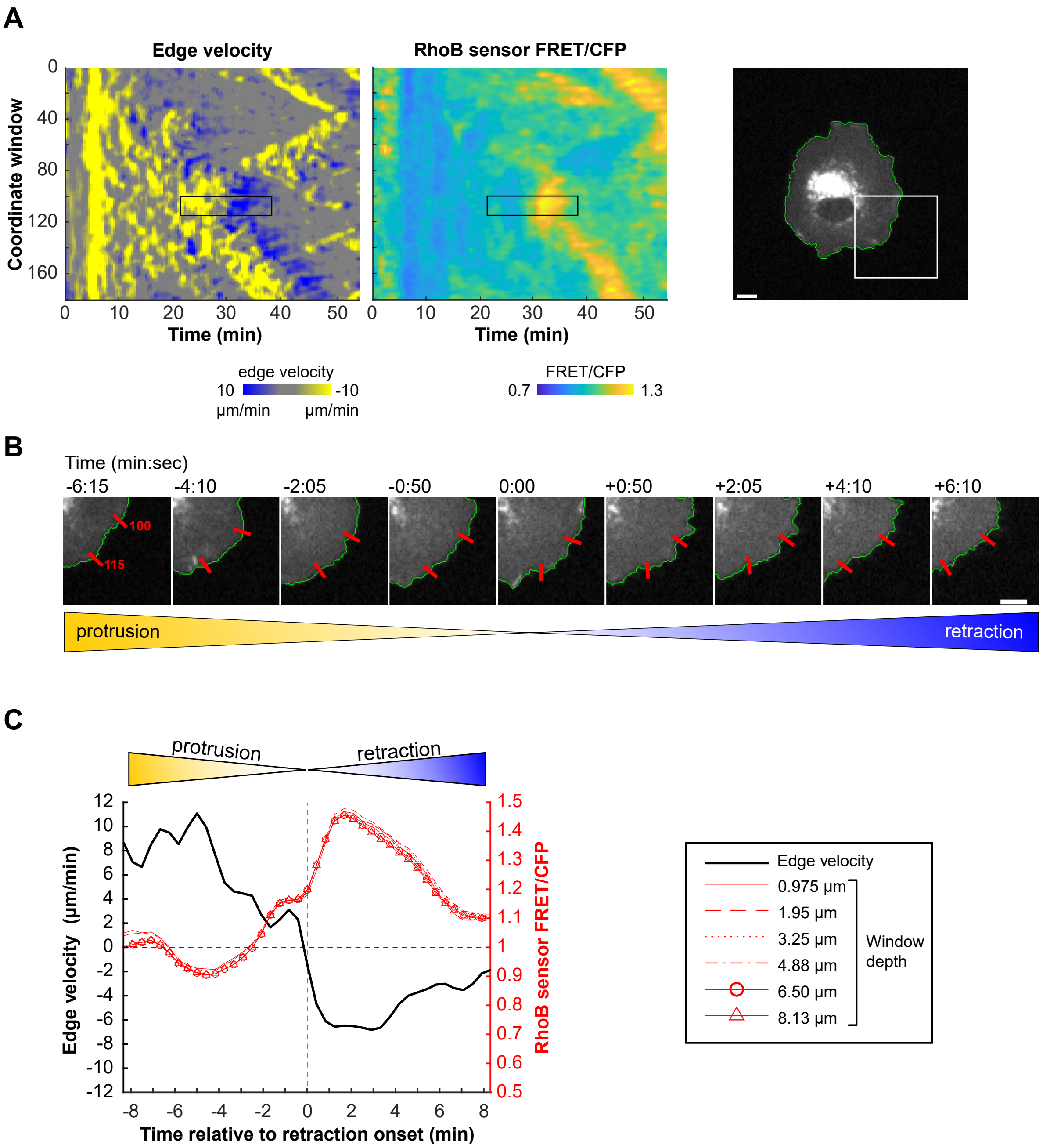

Figure S3. Edge velocity-Rho activity buildup analysis

(A) Edge velocity and Rho sensor FRET/CFP spatiotemporal heatmaps with region of interest outlined by a black rectangle. Black rectangle is 15 coordinate windows wide, approx. 8.33% of the cell perimeter, spanning a duration of 20 min. General region of analysis shown by white rectangle overlaid on cell outline on the right. Scale bar 10µm. **(B)** Time-lapse illustrating protrusion-retraction transition. Region of interest between coordinate windows 100 and 115 depicted by red ticks. Scale bar, 10µm. **(C)** Plot for the region of interest from **(A)** and **(B)** comparing average edge velocity to average RhoB sensor FRET/CFP activity per timepoint. Time=0 is placed at retraction onset, i.e., the transition from positive to negative edge velocity. RhoB sensor FRET/CFP per timepoint for varying window depths are plotted with different line styles.

A

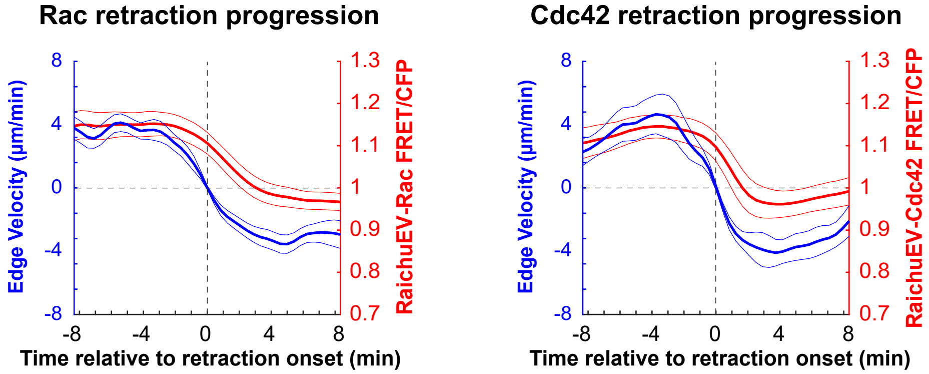

Figure S4. Rac and Cdc42 activity during protrusion-retraction transitions

(A) Edge velocity and RaichuEV-Rac (left) or Raichu EV-Cdc42 (right) FRET/CFP buildup plots displaying FRET/CFP at edge depths of 1.95µm during protrusion-retraction transitions. Time = 0 denotes retraction onset. Dataset means are bolded, bordered by ± 95% CI of the mean. RaichuEV-Rac: n =29 events, 4 biological replicates, RaichuEV-Cdc42: n =17 events, 3 biological replicates.

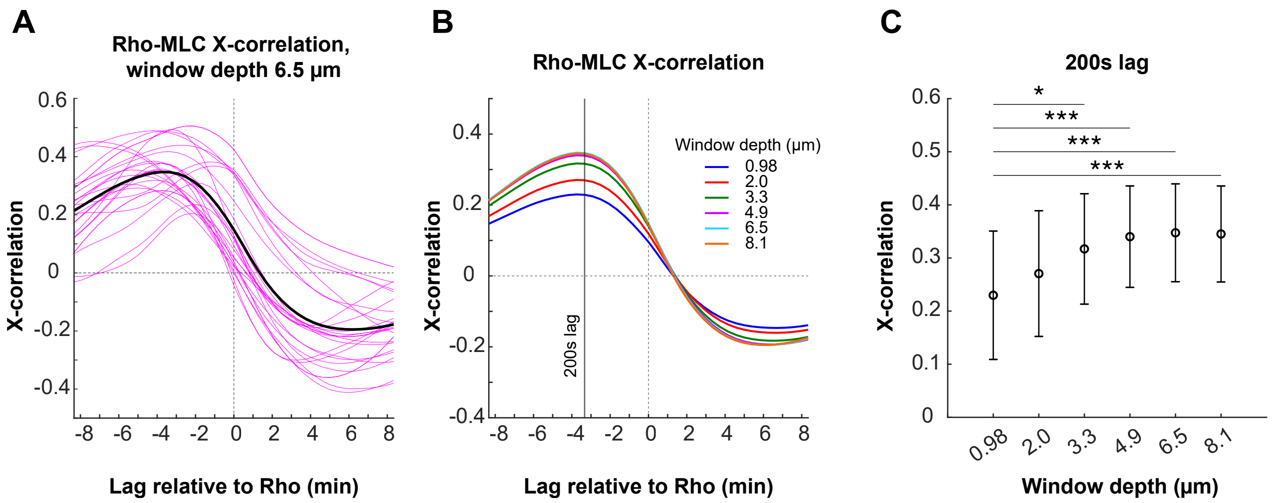

Figure S5. Cross-correlation between RhoB sensor activity and mRuby3-MLC signal
(A) Cross correlation between RhoB sensor FRET/CFP and mRuby3-MLC signal at an edge depth of 6.5µm. Pink lines represent results from individual cells, with the mean correlation bolded in black. n =27 cells from two biological replicates. **(B)** Average cross correlation between RhoB sensor FRET/CFP and mRuby3-MLC at window depths varying form 0.98 to 8.1µm. 200s lag, where all traces reach their maximum, is indicated by a vertical line. n=27 cells from two biological replicates. **(C)** Comparison of average correlation-coefficient at a lag of 200 s for all edge depths tested, i.e., a quantification of the difference of the traces in **(B)** at 200 s lag. Mean +/- 95% CI shown. n=27 cells from two biological replicates. *p<0.05, ***p<0.001, one-way ANOVA/Tukey-Kramer.

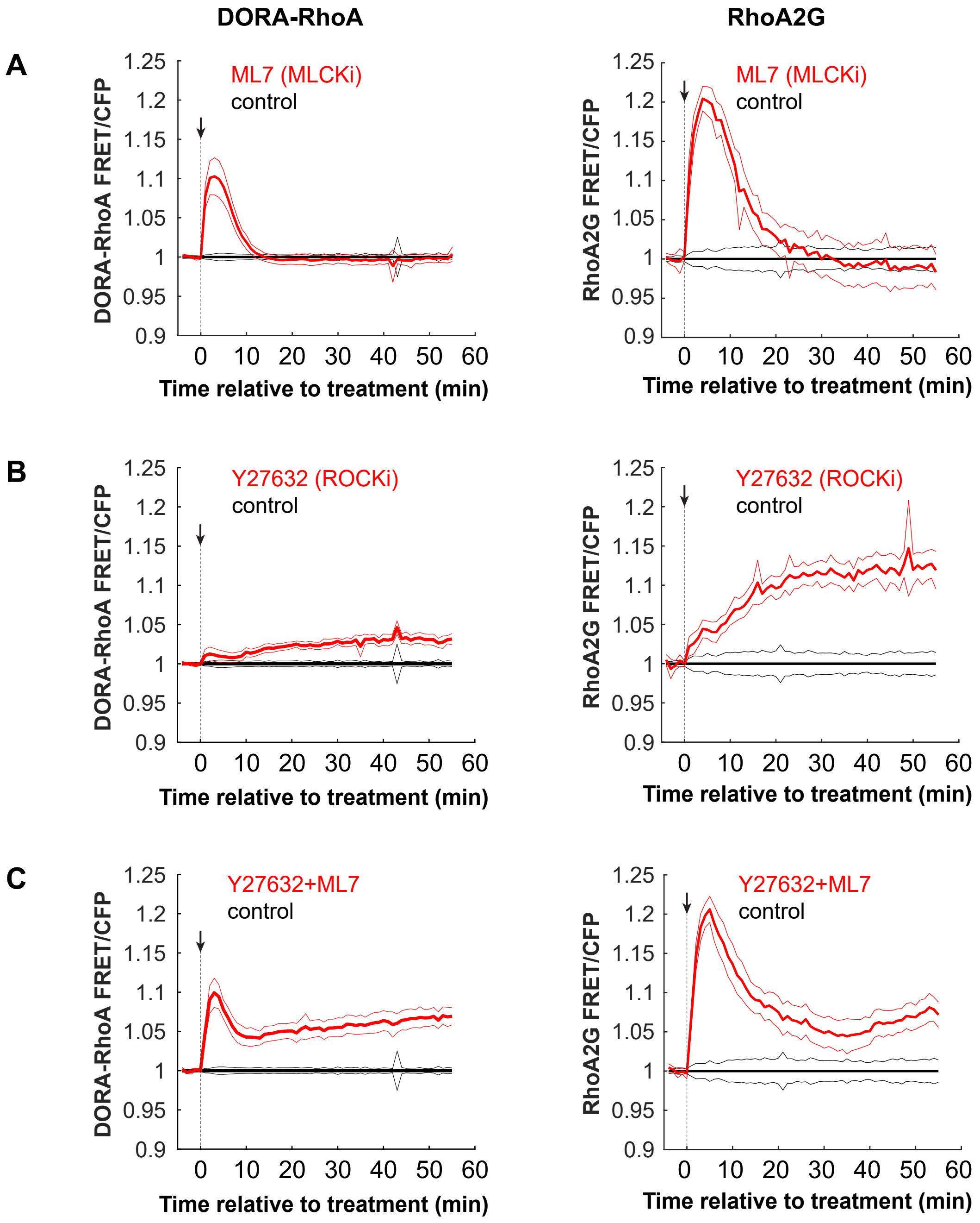

Figure S6. DORA-RhoA and RhoA2G responses to MLCK, ROCK inhibition
DORA-RhoA and RhoA2G activity responses to acute inhibition of **(A)** MLCK (ML7, 20μM), **(B)** ROCK (Y27632, 20μM), or **(C)** ROCK and MLCK (Y27632+ML7, both 20μM). Drug responses were normalized to control-treated samples. Bolded lines denote means, bordered by ± 95% CI. 16 fields of view (FOV) for each condition, from 4 technical, 2 biological replicates.

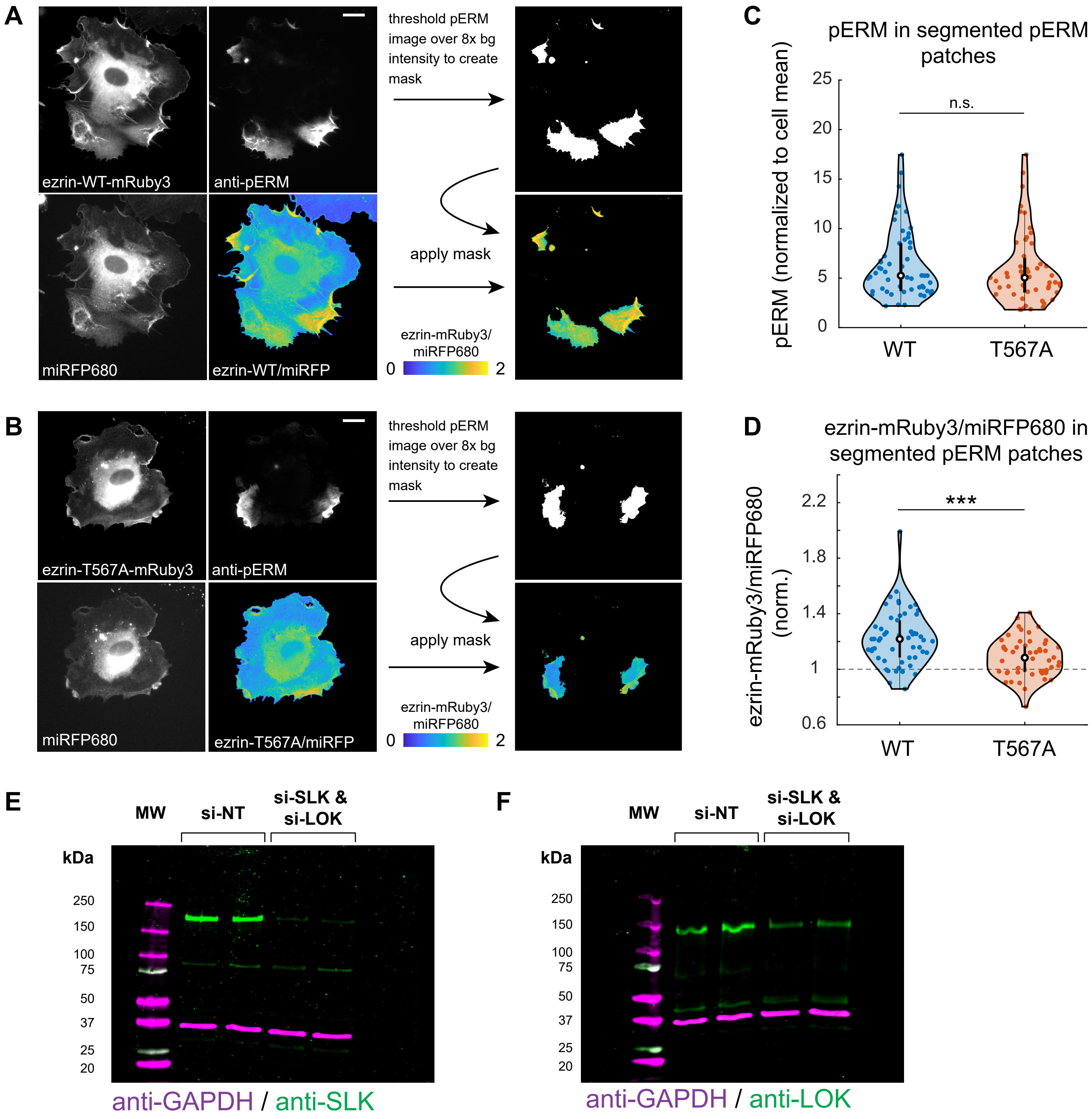

Figure S7. Validation of ezrin activation reporter and full blot scans

Illustrative examples of cells expressing **(A)** wildtype miRFP680-NES-p2A-ezrin-WT-mRuby3 or **(B)** the phosphomutant miRFP680-NES-p2A-ezrin-T567A-mRuby3. The normalized mRuby3/miRFP680 ratios are depicted. Cells had also been immunostained with anti-pERM antibody. Scale bars, 20µm. Cellular regions with increased pERM signals were identified by thresholding. mRuby3/miRFP680 ratios were measured in identified regions and normalized to average cellular mRuby3/miRFP680 ratios (Materials & Methods). **(C)** Normalized mean pERM intensities in thresholded pERM regions in cells expressing the WT or T567A variants. Median +/- 25th/75th percentile, and individual datapoints for n=54 (WT) or n=51 (T567A) cells from two biological replicates are shown. **(D)** Normalized average ezrin-mRuby3/miRFP680 ratios in thresholded pERM regions in cells expressing the WT or the T567A variants. Median ± 25th/75th percentile, and individual datapoints for n=54 (WT) or n=51 (T567A) cells from two biological replicates are shown. ***p<0.001, Mann-Whitney U-test. **(E,F)** Full scans of western blots shown in Fig. 6J.

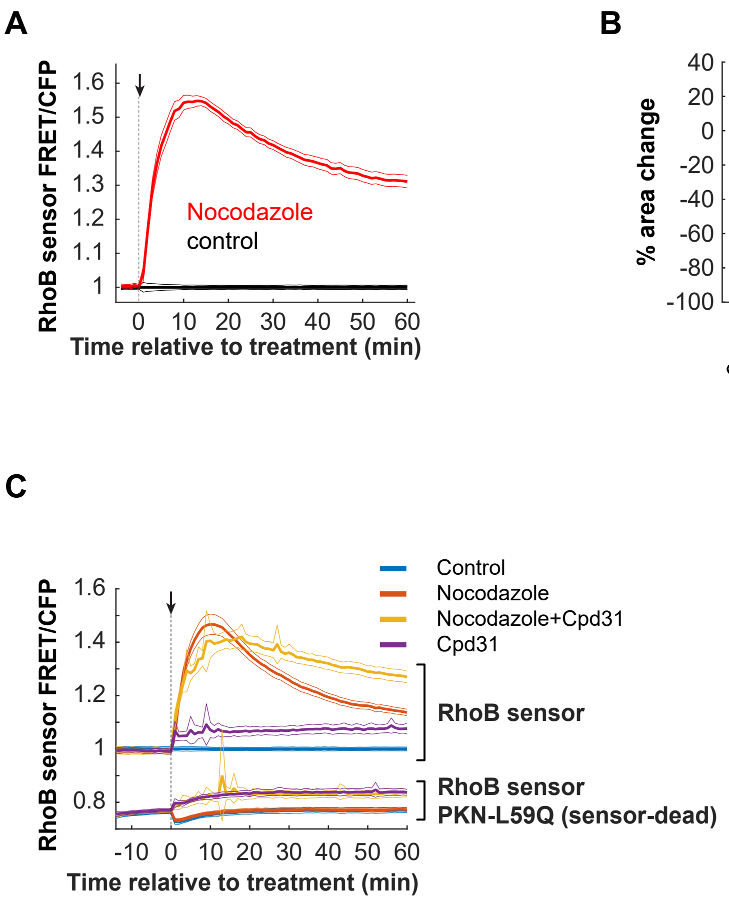

Figure S8. Additional RhoB sensor validations

(A) RhoB sensor activity response to acute nocodazole addition (10μM). Treated in red, normalized to control addition in black. Bolded lines denote mean, bordered by ± 95% CI. n=36 treated and control fields of view (FOV) each were analyzed, from 4 biological replicates. **(B)** Percent area changes of cells in control vs. nocodazole treated FOV, measured 30 min post nocodazole addition. Median ± 25th/75th percentile and data points for n=36 treated and control FOVs, from 4 biological replicates, are shown. ***p<0.001, Mann-Whitney U-test. **(C)** Responses of RhoB sensor and of a sensor-dead version of the RhoB sensor with RhoGTP binding-deficient PKN (PKN-L59Q) to control treatment or to treatment with nocodazole (10μM), Cpd31 (5μM) or nocodazole & Cpd31. All data were normalized to control-treated RhoB sensor responses. The presence of Cpd31 did not prevent nocodazole-induced Rho activation. The sensor-dead version of RhoB sensor PKN-L59Q showed minimal responses to all treatments. For each time-lapse sequence, the per frame average ratio metric FRET activity was calculated and the means of 16 time-lapse sequences (FOV) per condition are shown (bold), along with the 95% confidence intervals (thin). Four biological replicates, n=16 FOV for each condition.

Captions for Movies S1 to S17

Movie S1. HT-HUVEC stably expressing the RhoB sensor was imaged at 25sec intervals. Movie corresponds to Fig. 1A. YFP-FRET channel is shown, displayed at 15fps. Scale bar, 20µm.

Movie S2. Random motility of a HT-HUVEC stably expressing RaichuEV-Rac. *(Left)* Probe localization, *(right)*, FRET/CFP, scaling [0.7 1.3]. Acquired at 25sec intervals, displayed at 15fps. Scale bar, 20µm.

Movie S3. Random motility of a HT-HUVEC stably expressing RaichuEV-Cdc42. *(Left)* Probe localization, *(right)*, FRET/CFP, scaling [0.7 1.3]. Acquired at 25sec intervals, displayed at 15fps. Scale bar, 20µm.

Movie S4. Random motility of a HT-HUVEC stably expressing RhoB sensor. *(Left)* Probe localization, *(right)*, FRET/CFP, scaling [0.7 1.3]. Acquired at 25sec intervals, displayed at 15fps. Scale bar, 20µm.

Movie S5. Random motility of a HT-HUVEC stably expressing DORA-RhoA. *(Left)* Probe localization, *(right)*, FRET/CFP, scaling [0.7 1.3]. Acquired at 25sec intervals, displayed at 15fps. Scale bar, 20µm.

Movie S6. Random motility of a HT-HUVEC stably expressing RhoA2G. *(Left)* Probe localization, *(right)*, FRET/CFP, scaling [0.7 1.3]. Acquired at 25sec intervals, displayed at 15fps. Scale bar, 20µm.

Movie S7. Comparison of Rho activity as reported by the RhoB sensor and by the localization-based reporter dTomato-2xrGBD. dTomato-2xrGBD was ratioed over cytoplasmic miRFP680 to normalize for localization. Channels from left to right: dTomato-2xrGBD, miRFP680-NES, ratio dTomato-2xrGBD/miRFP680-NES scaling [0 2], RhoB sensor FRET/CFP, scaling [0.7 1.3]. Acquired at 25sec intervals, displayed at 15fps. Scale bar, 25µm.

Movie S8. HT-HUVEC stably expressing RhoB sensor during initial adhesion formation, spreading, and random motility. Retraction example in Fig. 3A, B occurs between t=20 and 30min. *(Left)* Probe localization, *(right)*, FRET/CFP, scaling [0.7 1.3]. Acquired at 25sec intervals, displayed at 15fps. Scale bar, 25µm.

Movie S9. HT-HUVEC stably expressing RhoB sensor exhibiting oscillatory RhoGTPase activity, characteristic of repeated protrusion-retraction cycles. *(Left)* Probe localization, *(right)*, FRET/CFP, scaling [0.7 1.3]. Acquired at 25sec intervals, displayed at 30fps. Scale bar, 20µm.

Movie S10. HT-HUVEC stably expressing RhoB sensor exhibiting a propagating wave of paired elevated RhoB activity and edge retraction. *(Left)* Probe localization, *(right)*, FRET/CFP, scaling [0.7 1.3]. Acquired at 25sec intervals, displayed at 30fps. Scale bar, 20µm.

Movie S11. HT-HUVEC stably co-expressing RhoB sensor and mRuby3-MLC undergoing random motility. Retraction example in Fig. 4A occurs between t=0min and t=23min. *(Left)* RhoB

sensor FRET/CFP, scaling [0.7 1.3], *(right)* mRuby3-MLC. Acquired at 15sec intervals, displayed at 30fps. Scale bar, 20µm.

Movie S12. HT-HUVEC stably co-expressing RhoB sensor and mRuby3-MLC, treated with 20µM ML7 at 5min. Channels from left to right: RhoB sensor localization, Rho sensor FRET/CFP [0.5 1.5], and mRuby3-MLC. Acquired at 1 min intervals, displayed at 15fps. Scale bar, 20µm.

Movie S13. HT-HUVEC stably co-expressing RhoB sensor and mRuby3-MLC, treated with 20µM Y27632 at 5min. Channels from left to right: RhoB sensor localization, Rho sensor FRET/CFP [0.5 1.5], and mRuby3-MLC. Acquired at 1min intervals, displayed at 15fps. Scale bar, 20µm.

Movie S14. HT-HUVEC stably co-expressing RhoB sensor and mRuby3-MLC. *(Left)* RhoB sensor FRET/CFP, scaling [0.7 1.3], *(center)* mRuby3-MLC. Movie displays movement of cell shown in Fig. 5A during 14min preceding fixation and anti-pERM immunostaining. (*right*) The same cell after anti-pERM immunostaining. Elevated pERM signal colocalizes with elevated RhoB sensor activity in retraction. Acquired at 1min intervals, displayed at 5fps. Scale bar, 20µm.

Movie S15. HT-HUVEC stably coexpressing RhoB sensor and miRFP680-NES-p2A-ezrin-mRuby3 during random motility. Channels from left to right: RhoB sensor localization, RhoB sensor FRET/CFP, scaling [0.7 1.3], ezrin-mRuby3/miRFP680 ratio, scaling [0.5 1.5]. Acquired at 25sec intervals, displayed at 15fps. Scale bar 20µm.

Movie S16. HT-HUVEC stably expressing the RhoB sensor (only localization is shown), either untreated (*left panels*) or treated with Cpd31 (5µM, 2h, *right panels)*. Acquired at 50sec intervals, displayed at 10fps. Scale bar 25µm.

Movie S17. HT-HUVEC stably expressing RhoB sensor were treated as depicted in Fig. 7I, movie corresponds to Fig. 7H. *(Left)* control, *(center left)* Nocodazole, 15µM, added at 15min and 45min. *(center right)* Nocodazole, 15µM, and Cpd31, 5µM, added at 15min. *(Right)* Nocodazole, 15µM, added at 15min, Cpd31, 5µM, added at 45min. RhoB sensor localization is shown. Acquired at 1min intervals, displayed at 15fps. Scale bar, 100µm.

Caption for Data S1

The datafile "**DataS1.xlsx**" contains **(1)** A list of all cell lines used with references to figure panels **(2)** raw data tables related to Figure 6 and **(3)** raw data tables related to Figure 7.
